# *Nrf2*-Linked Antioxidant and Metabolic Modulation by Dietary *Origanum vulgare* Essential Oil in Nile Tilapia Under Organophosphate Stress

**DOI:** 10.3390/biology15141117

**Published:** 2026-07-10

**Authors:** Yuniel Méndez-Martínez, Kerly Sánchez-Pacheco, Alison Reyes-Caracundo, Delia Olivares-Guadalupe, Edilmar Cortés-Jacinto

**Affiliations:** 1 Experimental Aquaculture Laboratory, Facultad de Ciencias Pecuarias, Universidad Técnica Estatal de Quevedo (UTEQ), Av. Quito Km. 1 1/2 via a Santo Domingo de los Tsáchilas, Quevedo 120301, Ecuador; ksanchezp2@uteq.edu.ec (K.S.-P.); areyesc4@uteq.edu.ec (A.R.-C.); dolivaresg@uteq.edu.ec (D.O.-G.); 2 Programa de Acuicultura, Centro de Investigaciones Biológicas del Noroeste (CIBNOR), Av. Instituto Politecnico Nacional #195, Playa Palo de Santa Rita Sur, La Paz 23096, Baja California Sur, Mexico; ecortes04@cibnor.mx

**Keywords:** *nrf2* signalling, antioxidant response, aquaculture nutrition, metabolic homeostasis, fish health, stress physiology

## Abstract

Phytobiotics are promising dietary tools to improve metabolic stability and physiological resilience against chemical stressors in aquaculture. This study evaluated *nrf2*-linked antioxidant and metabolic modulation by dietary *Origanum vulgare* essential oil (OEO) in Nile tilapia (*Oreochromis niloticus*) under malathion-induced organophosphate stress. Fish, with an initial weight of 5.76 ± 0.56 g, were distributed in three tanks per treatment, with 15 fish per tank, and fed diets containing 0 (control), 0.75, 1.50, 2.25 and 3.00 g kg^−1^ OEO for eight weeks, followed by exposure to malathion (7.04 mg L^−1^) for 96 h. OEO enhanced (*p* < 0.05) growth performance, feed utilisation, and survival after exposure, with the greatest productive and survival outcomes at 3.00 g kg^−1^. Fish that received the supplement also had lower (*p* < 0.05) lipid peroxidation, better antioxidant enzyme activity and a more favourable modulation of *nrf2*, *gpx* and *keap1* expression, together with biochemical and histological patterns that were consistent with a better condition of the liver and kidney. The heatmap and PCA supported a treatment-related separation, with 2.25 and 3.00 g kg^−1^ OEO showing the most favourable integrated physiological profiles. Dietary OEO was associated with antioxidant, metabolic and tissue-level resilience linked to modulation of *nrf2*-related transcriptional responses.

## Simple Summary

This study evaluated defence gene-linked antioxidant and metabolic modulation by dietary *Origanum vulgare* essential oil in Nile tilapia under malathion-induced organophosphate stress. Fish fed oregano essential oil grew better, used feed more efficiently and showed higher survival after pesticide exposure. They also showed stronger internal protection against stress, including a more favourable response of a gene involved in antioxidant defence, as well as better liver and kidney condition. Diets containing 2.25 and 3.00 g kg^−1^ oregano essential oil provided the best protection.

## 1. Introduction

Nile tilapia (*Oreochromis niloticus*) is one of the most important freshwater fish species in contemporary aquaculture because of its physiological plasticity, rapid growth, broad environmental tolerance and contribution to food security in tropical and subtropical regions [1,2]. In parallel with the continued expansion of aquaculture, there is a growing need for nutritional strategies capable of supporting physiological homeostasis, productive performance and resilience under increasingly complex farming conditions [3,4,5]. This need is reinforced by recent global trends showing that aquaculture has become the main driver of aquatic animal production, reaching 103 million tonnes in 2024 and accounting for 53% of global aquatic animal output [2,6]. Consequently, functional nutrition has received increasing attention as a strategy to modulate growth, oxidative balance, metabolism, immunity and tissue integrity in fish exposed to environmental, health-related or chemical stressors [7,8,9].

Among the chemical stressors of increasing relevance to inland aquaculture, organophosphate pesticides deserve special attention because of their widespread agricultural use and their potential entry into aquatic ecosystems through run-off, drainage and water recirculation. Malathion is a widely used organophosphate pesticide in agricultural systems and has classically been associated with cholinergic neurotoxicity, particularly through acetylcholinesterase inhibition [10,11]. However, in fish, the biological effects of organophosphates extend beyond cholinergic disruption and include systemic responses associated with oxidative stress, metabolic alteration, immunomodulation and tissue damage in functionally active organs [12,13]. Exposure to these compounds may increase reactive oxygen species generation, compromise antioxidant capacity, modify blood biochemical indicators and affect hepatorenal integrity, as well as modulate genes involved in cellular stress responses and peroxide detoxification processes [14,15,16,17,18].

On the other hand, the Kelch-like ECH-associated protein 1–nuclear factor erythroid 2-related factor 2/antioxidant response element pathway, commonly referred to as the Keap1–Nrf2/ARE pathway, is a central regulatory system for maintaining redox homeostasis under oxidative challenge [19,20]. Under basal conditions, Keap1 acts as a cytosolic repressor of Nrf2, promoting its ubiquitination and proteasomal degradation [21]. Under oxidative or electrophilic pressure, Nrf2 stabilisation may favour the transcriptional regulation of genes involved in antioxidant defence, detoxification and cell survival [19,20,22]. Although this pathway has been extensively characterised in mammals, growing evidence in aquatic organisms indicates that Nrf2-related signalling participates in fish responses to hypoxia, xenobiotics, oxidised oils, dietary bioactive compounds and other environmental stressors [21,23,24]. In this context, transcriptional markers related to the Keap1–Nrf2/ARE pathway provide a useful framework for evaluating redox-responsive modulation by functional feeds, while protein-level or functional assays remain necessary to directly confirm pathway activation [4,25].

Moreover, phytogenic feed additives have emerged as promising nutritional tools to improve fish resilience under intensive production and stress conditions [3,8,26]. Essential oils are of particular interest because they represent complex mixtures of bioactive compounds with antioxidant, antimicrobial, anti-inflammatory, digestive and immunomodulatory properties [9,27,28,29]. *Origanum vulgare* essential oil (OEO) is especially relevant because some chemotypes contain carvacrol, thymol, γ-terpinene and *p*-cymene, compounds that have been associated with redox regulation, microbial control and modulation of inflammatory and immune responses [30,31,32]. However, given its complex chemical composition, the biological effects of OEO should be interpreted as the integrated action of the essential oil matrix rather than as the isolated activity of individual constituents.

In farmed fish, dietary OEO supplementation has shown functional effects that vary according to species, dose and experimental model. In Nile tilapia (*O. niloticus*), improvements in growth, feed efficiency, haemato-biochemical responses and immunity under intensive culture conditions have been reported [29,33,34,35]. In other farmed species, including channel catfish (*Ictalurus punctatus*), common carp (*Cyprinus carpio*), European sea bass (*Dicentrarchus labrax*) and red tilapia hybrids, OEO or oregano-derived supplementation has been associated with antioxidant responses, stress resistance, intestinal morphometry, hepatorenal condition and metabolic status [27,28,36,37].

Although meta-analytical evidence supports the potential of phytogenic supplements to improve growth, immunity and disease resistance in aquaculture, evidence remains limited on whether dietary OEO supplementation is associated with coordinated antioxidant, metabolic, transcriptional and tissue-level responses in fish exposed to organophosphate-induced stress. In particular, few studies have integrated oxidative status, blood biochemical indicators, *nrf2*-related gene expression and hepatorenal histology within the same experimental framework during pesticide exposure.

This limitation makes it difficult to determine whether the responses associated with OEO supplementation reflect a general antioxidant effect or a broader physiological modulation involving redox-related transcriptional pathways and tissue preservation. Consequently, it was hypothesised that dietary OEO supplementation would be associated with improved physiological resilience and a more favourable antioxidant, metabolic, transcriptional and hepatorenal profile in Nile tilapia exposed to an acute malathion challenge. Therefore, this study evaluated whether graded dietary supplementation with OEO is associated with changes in growth performance, metabolic status, antioxidant capacity, lipid peroxidation, *nrf2*-related gene expression and hepatorenal integrity in Nile tilapia under malathion-induced organophosphate stress.

## 2. Materials and Methods

### 2.1. Study Site

The experiment was carried out in an indoor facility at the Aquaculture Laboratory of the Universidad Técnica Estatal de Quevedo (UTEQ), located in Quevedo, Los Ríos province, Ecuador. The geographical coordinates are 01°06′13″ S and 79°29′22″ W, at an altitude of 73 m above sea level.

### 2.2. Extraction and Storage of OEO

The OEO was obtained from dried leaves and inflorescences by hydrodistillation, following previously described protocols [38,39]. For this purpose, the dried and fragmented plant material was placed in a round-bottom flask with distilled water, maintaining a plant material:water ratio of 1:10 (*w*/*v*). The flask was connected to a Clevenger-type hydrodistillation unit equipped with a condenser (Borosil Scientific Ltd., Mumbai, India), and the system was heated until gentle boiling was achieved. The extraction process was maintained for 3 h after the onset of boiling. Subsequently, the OEO was recovered using a Pasteur pipette (Deltalab S.L., Barcelona, Spain), dried over anhydrous sodium sulphate (Na_2_SO_4_) and transferred to tightly sealed amber glass vials. The OEO was stored at 4 °C until use.

### 2.3. Chemical Characterisation of OEO

The volatile profile of OEO was characterised by GC-MS using an Agilent 7890B/5977A GC-MS system (Agilent Technologies, Santa Clara, CA, USA) [40,41]. The oil was diluted 1:100 (*v*/*v*) in HPLC-grade *n*-hexane, homogenised, filtered through a 0.22 µm PTFE syringe filter and injected into a DB-5MS capillary column (30 m × 0.25 mm i.d. × 0.25 µm). Helium was used as the carrier gas at 1.1 mL min^−1^, and 1 µL of the diluted sample was injected in split mode (1:50) at 260 °C. The oven programme was set at 60 °C for 5 min, increased to 250 °C at 5 °C min^−1^ and held for 10 min. The MS operated in EI mode at 70 eV, with ion source and transfer line temperatures of 230 °C and 240 °C, respectively, and spectra were acquired in full scan mode from *m*/*z* 35 to 450. Compounds were identified by matching their mass spectra with NIST/Wiley libraries (https://webbook.nist.gov/chemistry/, accessed on 10 January 2026) and by comparing experimental linear retention indices, calculated using a homologous series of *n*-alkanes [42,43].

### 2.4. Formulation and Preparation of Experimental Diets

Five practical diets were formulated (Table 1) to be isonitrogenous and isolipidic, differing only in the dietary inclusion level of OEO, which was incorporated at 0 (control), 0.75, 1.50, 2.25 and 3.00 g kg^−1^ diet. The OEO doses used in our bioassay were established based on previous studies in teleost fish [27,28,36,37]. The graded inclusion levels were selected to evaluate a biologically plausible dose–response gradient, ranging from an unsupplemented control diet to a relatively high but practical inclusion level, without exceeding ranges previously reported as functional in fish. Diet formulation was carried out using LINDO software (LINDO Systems Inc., v6, Chicago, IL, USA).

Before diet manufacture, the dry macro-ingredients were ground when necessary and passed through a 250 µm sieve to improve particle uniformity. All ingredients were accurately weighed using a digital balance (OHAUS Corporation, Parsippany, NJ, USA). The macro-ingredients were first blended until a homogeneous basal mixture was obtained, whereas the micro-ingredients were premixed separately to ensure an even distribution before their incorporation into the main mixture.

For each experimental diet, the corresponding amount of OEO was previously dispersed in fish oil and then gradually added to the dry mixture [44]. After lipid incorporation, water was added at approximately 30% of the ingredient weight to obtain a suitable dough for pellet formation. The resulting mixture was processed twice through a meat grinder (Oster, Sunbeam Products Inc., Boca Raton, FL, USA) to produce 2 mm pellets. The pellets were dried in a forced-air oven (Memmert GmbH + Co. KG, Schwabach, Germany) at 45 °C for 8 h, packed in plastic bags and stored at 4 °C until use.

### 2.5. Chemical Analysis of Diets

The proximate composition of the experimental diets (Table 1) was analysed according to AOAC [45]. Samples were dried at 105 °C to constant weight for determination of moisture content and incinerated at 550 °C for 8 h in a muffle furnace to determine ash. Total nitrogen was multiplied by 6.25 to determine crude protein. Nitrogen was determined by the Kjeldahl method. The ether extract was estimated by solvent extraction with petroleum ether using a Soxtec system. Crude fibre was determined according to the Weende procedure with sequential acid and alkaline digestions using 1.25% H_2_SO_4_ and 1.25% NaOH, respectively. Nitrogen-free extract was estimated by difference. All determinations were conducted in triplicate. Digestible energy was calculated theoretically using conversion factors of 4.25 kcal g^−1^ for animal protein, 3.8 kcal g^−1^ for vegetable protein, 8.0 kcal g^−1^ for lipids, 2.0 kcal g^−1^ for carbohydrates from legumes and 3.0 kcal g^−1^ for carbohydrates from non-legume ingredients, according to Ramanathan et al. [46].

### 2.6. Experimental Design and Rearing Conditions

Juvenile Nile tilapia, *O. niloticus* (5.76 ± 0.56 g), were obtained from Natural Fish S.A.S. in Yaguachi Canton, Guayas, Ecuador, and transported to the Aquaculture Laboratory of UTEQ.

Fish were acclimated to laboratory rearing conditions for seven days before the trial. The experiment was conducted under a completely randomised design. A total of 225 juvenile Nile tilapia were used and randomly distributed into 15 plastic tanks of 50 L, corresponding to five dietary treatments (see Section 2.4), with three replicate tanks per treatment and 15 fish per tank. Treatments were randomly assigned to the tanks, and all tanks were maintained under the same husbandry conditions throughout the eight-week feeding trial. The tank was considered the experimental unit for interpreting treatment effects.

Dissolved oxygen was measured using an OHAUS ST400D Starter 400D digital oximeter (OHAUS Corporation, Parsippany, NJ, USA), temperature with a mercury thermometer (0–50 °C), and pH and ammonium with a colorimetric kit (API Saltwater Master, Chalfont, PA, USA). Fish were maintained on a natural photoperiod of 12 h light: 12 h dark. Daily monitoring of water quality was performed with mean values of 6.5 ± 0.45 mg L^−1^ dissolved oxygen, 27.75 ± 0.32 °C temperature, 0.11 ± 0.03 mg L^−1^ ammonium and 7.01 ± 0.04 pH. These values were within the recommended ranges for tilapia culture [47,48].

Tanks were syphoned daily before feeding to remove faeces and residual feed, and the removed water was replaced. Fish were fed twice daily, at 09:00 and 17:00 h, until apparent satiation. Uneaten feed was collected the following morning, concentrated on Whatman No. 1 filter paper (Cytiva, Marlborough, MA, USA) using a vacuum pump and dried at 50 °C for 18 h in a forced-air oven [49,50]. Apparent feed intake was estimated for each replicate tank as the difference between the dry feed offered and the dried uneaten feed recovered. Feed allocation was adjusted weekly based on apparent feed intake to minimise feed residues.

### 2.7. Sample Collection

At the end of the eight-week feeding trial, fish were fasted for 24 h and subsequently anaesthetised with eugenol (40 mg L^−1^). After individual body weight and total length had been recorded for all fish, five juveniles per replicate tank (*n* = 15 per treatment) were randomly selected for baseline sampling before malathion exposure. Blood was collected from these same fish by caudal puncture using sterile disposable 1 mL syringes for biochemical analyses. Blood samples were centrifuged at 3000 rpm for 10 min at 4 °C, and the resulting plasma was stored at −20 °C until analysis. After blood collection, the same fish were euthanised with an overdose of eugenol (80 mg L^−1^). Death was confirmed by the absence of opercular movements, loss of the righting reflex and lack of response to tactile and ocular stimuli. Thereafter, the liver and head kidney from the same fish were carefully dissected. From each sampled fish, portions of the liver and head kidney were fixed in 10% neutral-buffered formalin for histological evaluation. In addition, a separate portion from the same liver of each sampled fish was preserved in RNAlater^®^ under RNase-free conditions for hepatic gene expression analysis.

The remaining fish in each replicate tank, namely 10 fish per tank and 30 fish per treatment, were retained for the malathion exposure phase. At the end of the 96 h malathion exposure, five exposed fish per replicate tank (*n* = 15 per treatment) were randomly selected, and the same procedures for anaesthesia, blood collection, tissue dissection and sample preservation were repeated for post-exposure analyses.

### 2.8. Fish Growth Performance

Mathematical formulae were used to determine specific growth rate, weight gain, feed conversion ratio, apparent feed intake, condition factor and survival rate. These are detailed below:

Weight Gain (g) = [*Wx* − *Wi*] (1)

Specific Growth Rate = 100 × [(ln*Wx* − ln*Wi*)/*t*] (2)

Condition Factor = 100 × [*Wx*/*Lx*^3^] (3)

Feed Conversion Ratio = *Tfc*/*Twc* (4)

Survival Rate (%) = 100 × [final number of fish/initial number of fish](5)

Apparent feed intake (g fish^−1^day^−1^) = [*Dfo* − *Dufr*]/[*Nft* × *t*] (6)

where *t*: duration of experiment (days), *Lx*: final body length (cm), *Wx*: final body weight (g), *Wi*: initial body weight (g), *Tfc*: total feed consumed (g, dry weight), and *Twc*: total weight gain (g, wet weight), *Dfo*: dry feed offered (g), *Dufr*: dried uneaten feed recovered (g), and *Nft*: number of fish in the tank.

### 2.9. Organophosphate-Induced Stress

After eight weeks of experimental feeding, fish from the five dietary treatments previously described in Section 2.4 [0 (control); 0.75; 1.50; 2.25 and 3.00 g kg^−1^ OEO] were used for the acute malathion exposure phase. For this purpose, 10 fish per replicate tank, equivalent to 30 fish per treatment, were maintained and exposed for 96 h to a nominal malathion concentration of 7.04 mg L^−1^. This concentration was selected to apply a severe and standardised acute stress, following the general approach of acute toxicity tests in fish based on the estimation of the LC_50_-96 h [51] and previous studies of acute malathion toxicity in fish [12]. This allowed the physiological response of fish previously fed the experimental diets to be evaluated under intense organophosphate exposure. During the exposure, fish were maintained under controlled water-quality conditions.

The exposure was conducted under a semi-static system, with water renewal and preparation of fresh malathion solutions every 24 h. The physicochemical parameters of the water were monitored throughout the trial. Dead fish were removed immediately, and survival was recorded during the exposure period and calculated at the end of 96 h.

The nominal malathion concentration used in this assay corresponded to the LC_50_-96 h previously established through an independent concentration–response bioassay. Mortality data obtained from this bioassay were analysed using the Probit method. A detailed description of the LC_50_-96 h bioassay and the Probit model curve are included in the Supplementary Materials (Figure S1).

### 2.10. Blood Biochemistry

Plasma biochemical parameters were determined using LiquiColor^®^ commercial kits (Society for Biochemistry and Diagnostics, Wiesbaden, Germany), following the manufacturer’s instructions and in triplicate. All reactions were incubated at 37 °C and absorbance was measured using a BTS-350 semi-automatic spectrophotometer (BioSystems, S.A., Barcelona, Spain) with appropriate wavelength calibration. Total protein (g dL^−1^) was quantified by the biuret method (546 nm, after 10 min incubation); albumin (g dL^−1^) by the BCG dye-binding method (630 nm, 5 min); glucose (mg dL^−1^) by GOD-PAP (500 nm, 10 min); triglycerides (mg dL^−1^) by the GPO-PAP method (546 nm, 10 min); and total cholesterol (mg dL^−1^) by CHOD-PAP (500 nm, 10 min). Urea (mg dL^−1^) was evaluated by the urease–GLDH method (340 nm, 5 min), while creatinine (mg dL^−1^) was measured using the kinetic Jaffé method with dual readings at 492 nm after 30 and 120 s, respectively [52,53]. The activities of aspartate aminotransferase (AST), alanine aminotransferase (ALT) and alkaline phosphatase (ALP) enzymes were determined by the method of Bergmeyer et al. [54]. The samples were incubated for 15 min at 37 °C for AST, 5 min at 37 °C for ALT and 6 min at 35 °C for ALP. Absorbance measurements were taken for 3 min at an absorbance (ABS) of 340 nm for AST and ALT and for 2 min at 405 nm ABS for ALP using a spectrophotometer [55]. Enzyme activity was expressed as U L^−1^. All measurements were performed in triplicate to ensure analytical precision and reproducibility.

### 2.11. Antioxidant Capacity

Antioxidant enzyme activities were determined by colourimetric kinetic assays using commercial reagents (Randox Laboratories Ltd., Crumlin, United Kingdom) and standard methodologies. Superoxide dismutase (SOD) activity was assayed by inhibition of the reduction in iodonitrotetrazolium chloride (INT) by superoxide radicals generated via the xanthine/xanthine-oxidase system; absorbance was read at 505 nm [56,57]. Glutathione peroxidase (GPx) activity was quantified by monitoring the decrease in NADPH at 340 nm in the presence of reduced glutathione, cumene hydroperoxide, and glutathione reductase [58]. Catalase (CAT) activity was determined by the Aebi spectrophotometric method [58], based on the decomposition of hydrogen peroxide; absorbance at 240 nm was monitored at 25 °C for 3 min. Enzyme activities were expressed as U mL^−1^. For malondialdehyde (MDA) analysis, the TBARS method based on the reaction with thiobarbituric acid was used, with spectrophotometric reading at 532 nm. MDA concentration was calculated using a standard curve of 1,1,3,3-tetraethoxypropane [59]. All analyses were conducted in triplicate.

### 2.12. Gene Expression

Total RNA was extracted from approximately 50 mg of liver tissue per sample using GENEzol™ Reagent (Geneaid Biotech Ltd., New Taipei City, Taiwan). RNA concentration and purity were determined spectrophotometrically, whereas RNA integrity was verified by agarose gel electrophoresis. In addition, no-reverse-transcriptase controls were included to exclude genomic DNA contamination, in accordance with RT-qPCR quality recommendations [60,61]. The relative expression of *nrf2*, *keap1*, and *gpx* was quantified by RT-qPCR using TOPreal™ SYBR Green qPCR PreMIX (Enzynomics Co., Ltd., Daejeon, Republic of Korea) in a Rotor-Gene Q thermocycler (QIAGEN GmbH, Hilden, Germany). Each 20 µL reaction contained 10 µL of master mix, 0.6 µL of each primer, 1 µL of cDNA template, and nuclease-free water to complete the final volume. Primer characteristics are presented in Table 2.

The internal reference genes *18S rRNA* and *ef1α* were selected based on their reported stability in Nile tilapia [62,63] and were further evaluated under the present experimental conditions using geNorm. Both reference genes showed M values below the recommended geNorm stability threshold of 1.5, supporting their use for RT-qPCR normalisation. The amplification program consisted of an initial activation step at 95 °C for 15 min, followed by 40 cycles of 95 °C for 10 s, primer-specific annealing for 15–20 s, and extension at 72 °C for 10–25 s. A melting-curve analysis was performed at the end of each run to confirm amplicon specificity. All reactions were carried out in technical triplicate, and relative gene expression was calculated using the 2^−ΔΔCt^ method [60].

### 2.13. Histology

Liver and kidney samples were fixed in 10% neutral-buffered formalin to preserve tissue architecture before routine histological processing. After fixation, the tissues were dehydrated through an ascending ethanol series (70–100%), cleared, embedded in paraffin and sectioned at 5 µm using a rotary microtome. The sections were mounted on glass slides and stained with haematoxylin and eosin (H&E) for the general assessment of hepatic and renal tissue organisation. Histological evaluation was performed using a light microscope coupled to a digital colour camera. Representative micrographs were captured from each experimental group, and digital image analysis was carried out using Image Scion 4.0.2 software. The histological evaluation and image analysis were performed by observers blinded to the dietary treatment and exposure condition.

### 2.14. Statistical Analysis

Data were analysed considering the replicate tank as the experimental unit. Normality and homogeneity of variances were verified using the Kolmogorov–Smirnov and Levene tests, respectively. When necessary, variables expressed as proportions or percentages were transformed using the arcsine square-root transformation [y′ = arcsin(√*p*)]. Treatment effects were evaluated using one-way analysis of variance (ANOVA), considering dietary treatment as the fixed factor. When significant differences were detected, Duncan’s post hoc test was applied. Differences were considered significant at *p* < 0.05. These analyses were performed using Minitab^®^ Statistical Software, version 21 (Minitab LLC, State College, PA, USA).

Exploratory multivariate analyses were performed to evaluate integrated response patterns among treatments before and after malathion exposure. Prior to multivariate analysis, the data matrix was autoscaled by mean centring and scaling to unit variance, in order to allow the joint comparison of variables measured in different units. Heatmaps were generated from the autoscaled matrix. Principal component analysis (PCA) was performed using the correlation matrix, and variable loadings were examined to identify the biomarkers that contributed mainly to treatment separation.

Global multivariate differences among dietary treatments were evaluated using permutational multivariate analysis of variance (PERMANOVA), based on Euclidean distances calculated from the autoscaled data matrix. The model included dietary treatment as the fixed factor and the replicate tank as the experimental unit: distance matrix ~ dietary treatment. A total of 9999 permutations was used. Homogeneity of multivariate dispersion among treatments was evaluated using PERMDISP, with the aim of verifying that the results of the permutational analysis were not explained by marked differences in within-group dispersion. In accordance with the experimental design based on replicate tanks, multivariate results were interpreted as exploratory and complementary to the univariate analyses. Heatmap, PCA, PERMANOVA and PERMDISP analyses were performed in RStudio^®^ version 2023.06.1+524 (RStudio, Boston, MA, USA), using the *pheatmap*, *FactoMineR* and *vegan* packages.

## 3. Results

### 3.1. Phytochemical Profile of OEO

**Figure 1:**
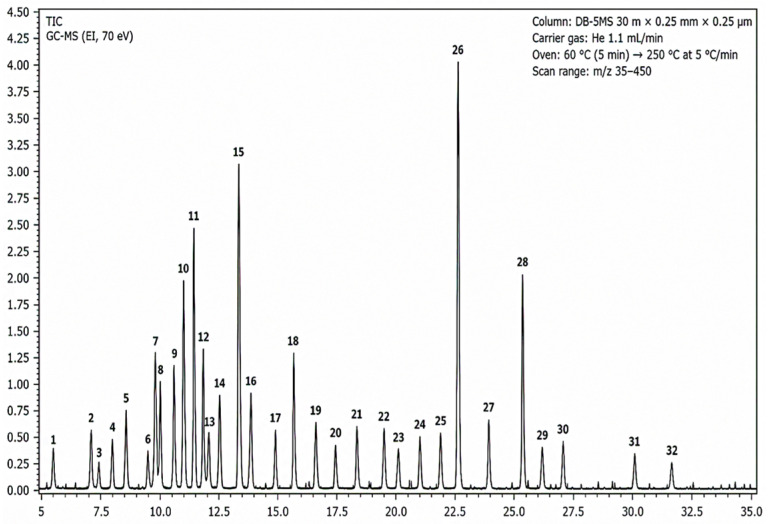
GC-MS total ion chromatogram of OEO, showing retention time (min) on the x-axis and signal intensity (×100,000) on the y-axis. (1) α-thujene; (2) α-pinene; (3) camphene; (4) sabinene; (5) β-pinene; (6) 1-octen-3-ol; (7) myrcene; (8) α-phellandrene; (9) Δ-3-carene; (10) α-terpinene; (11) *p*-cymene; (12) limonene; (13) β-phellandrene; (14) (Z)-β-ocimene; (15) γ-terpinene; (16) cis-sabinene hydrate; (17) terpinolene; (18) linalool; (19) trans-sabinene hydrate; (20) borneol; (21) terpinen-4-ol; (22) α-terpineol; (23) carvacrol methyl ether; (24) thymoquinone; (25) thymol; (26) carvacrol; (27) carvacryl acetate; (28) (E)-β-caryophyllene; (29) α-humulene; (30) caryophyllene oxide; (31) epi-α-muurolol; (32) α-eudesmol.

The phytochemical profile of OEO (Figure 1) revealed a volatile matrix dominated by terpenic compounds, with 32 constituents identified by GC-MS. In the TIC chromatogram, the highest-intensity peaks corresponded mainly to carvacrol (peak 26; RT = 22.67 min), γ-terpinene (peak 15; RT = 13.34 min), *p*-cymene (peak 11; RT = 11.36 min) and (E)-β-caryophyllene (peak 28; RT = 25.33 min), which was consistent with the percentage distribution reported in Table 3, where carvacrol was the predominant compound (61.6%), followed by γ-terpinene (9.0%), *p*-cymene (6.9%) and (E)-β-caryophyllene (5.1%). In addition, the occurrence of α-terpinene, limonene, myrcene, linalool, thymol, carvacryl acetate, caryophyllene oxide and other minor constituents confirmed that the OEO did not represent a single chemical fraction, but rather a complex mixture composed of monoterpene hydrocarbons, oxygenated monoterpenes, phenolic monoterpenes and sesquiterpenes.

### 3.2. Growth Performance and Survival

Table 4 shows that the growth performance of *O. niloticus* was significantly affected by dietary OEO levels after eight weeks (*p* < 0.05). Initial weight did not differ among treatments (*p* > 0.05). The highest values of final weight (28.02 g), final length (132.11 mm), weight gain (22.23 g), specific growth rate (2.82% day^−1^), and protein efficiency ratio (1.93) were observed in fish fed 3.00 g kg^−1^ OEO, whereas the control treatment showed the lowest values. The feed conversion ratio decreased significantly with increasing dietary OEO inclusion, reaching the lowest value at 3.00 g kg^−1^.

The apparent feed intake and condition factor did not show significant differences among treatments (*p* = 0.0721, 0.0561), respectively. Likewise, survival was 100% in all groups during the eight weeks before exposure to the toxicant. However, after malathion exposure, survival was significantly affected by dietary OEO supplementation (*p* = 0.0013), with the lowest value observed in the exposed control group (0 g kg^−1^ OEO + malathion; 53.33%) and the highest value observed in fish fed 3.00 g kg^−1^ OEO (76.67%).

### 3.3. Metabolic Biomarkers

Table 5 shows that the metabolic biomarkers of *O. niloticus* varied significantly among treatments before and after malathion exposure (*p* < 0.05). Before exposure, glucose and total protein tended to increase with supplementation, reaching their highest values at 3.00 g kg^−1^ (60.01 mg dL^−1^ and 3.69 g dL^−1^, respectively). In contrast, bilirubin, urea, and creatinine showed a general decreasing trend, with the lowest values observed at 2.25–3.00 g kg^−1^. Meanwhile, AST, ALT, and ALP increased progressively, also reaching their maximum values at 3.00 g kg^−1^.

After exposure, significant differences were also found among treatments (*p* < 0.05); however, the response pattern changed. Glucose, bilirubin, urea, creatinine, and the enzymes AST, ALT, and ALP were highest in the control group (0 g kg^−1^ OEO + malathion), whereas supplemented treatments, especially 2.25 and 3.00 g kg^−1^, showed lower values. In addition, cholesterol decreased with increasing OEO inclusion, reaching its minimum value at 3.00 g kg^−1^, while total protein was highest in this same treatment.

### 3.4. Antioxidant Capacity

Table 6 shows that the antioxidant capacity of *O. niloticus* was significantly affected by the dietary levels of *O. vulgare* OEO before and after malathion exposure (*p* < 0.05). Before exposure, SOD, CAT, GPx and MDA differed significantly among treatments. The highest SOD values were recorded at 1.50 and 3.00 g kg^−1^, whereas CAT reached its maximum value at 2.25 g kg^−1^ and GPx was higher at 1.50, 2.25 and 3.00 g kg^−1^. In contrast, MDA decreased progressively with increasing OEO inclusion, reaching the lowest value at 3.00 g kg^−1^. SOD, CAT, GPx and MDA differed significantly among treatments after exposure. SOD activity was highest at 2.25 g kg^−1^, CAT at 3.00 g kg^−1^ and GPx at 1.50–2.25 g kg^−1^ while MDA levels were higher in control and lower in supplemented treatments with minimum value at 3.00 g kg^−1^.

### 3.5. Relative Expression of Genes

**Figure 2:**
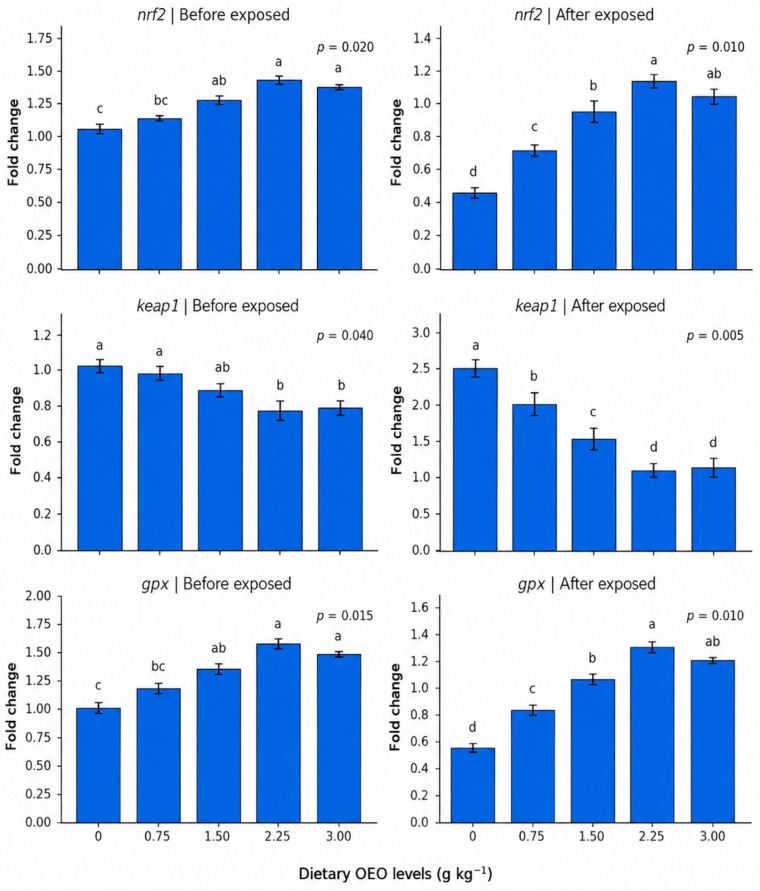
Relative gene expression in *O. niloticus* fed diets supplemented with OEO for 8 weeks and subsequently exposed to malathion-induced stress for 96 h. Results are reported as means ± standard error of 3 groups per treatment (*n* = 3). Different letters (abcd) indicate significant differences (*p* < 0.05).

Figure 2 shows that the relative expression of *nrf2*, *keap1* and *gpx* in *O. niloticus* was significantly affected by supplementation with OEO before and after malathion exposure (*p* < 0.05). Before exposure, *nrf2* increased from 1.05 in the control group to 1.45 at 2.25 g kg^−1^, while *gpx* increased from 1.00 to 1.58 in the same treatment. In contrast, *keap1* decreased from 1.03 in the control group to 0.78 at 2.25 g kg^−1^.

After exposure, the control group (0 g kg^−1^ OEO + malathion) showed the least favourable pattern, with the lowest expression levels of *nrf2* (0.44) and *gpx* (0.55), and the highest expression of *keap1* (2.52). In contrast, supplemented fish, especially those fed 2.25 and 3.00 g kg^−1^, showed a more protective response, with higher *nrf2* expression (1.15 and 1.05, respectively) and *gpx* expression (1.32 and 1.25, respectively) in addition to lower *keap1* values (1.10 and 1.15, respectively).

### 3.6. Histology of Liver

**Figure 3:**
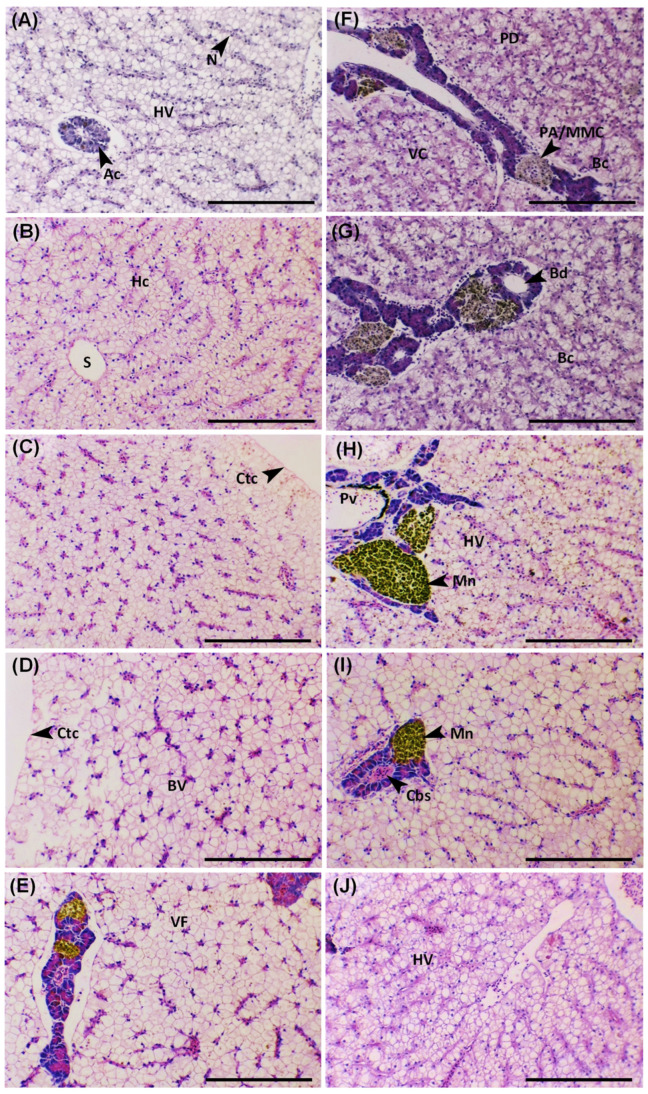
Liver histological micrographs of *O. niloticus* fed diets supplemented with 0, 0.75, 1.50, 2.25 and 3.00 g kg^−1^ OEO for eight weeks before exposure (**A**–**E**) and after 96 h of malathion-induced stress under the same dietary treatments (**F**–**J**), respectively. Haematoxylin and eosin (H&E) staining. Scale bar: 100 µm. Total magnification: 100×. Abbreviations: Ac, pancreatic acinar cells; Bc, blood congestion; Bd, bile duct; BV, blood vessel; Cbs, congested blood sinusoids; Ctc, connective tissue capsule; Hc, hepatocytes; HV, hepatocyte vacuolisation; Mn, melanomacrophages; N, nuclei; PA/MMC, pigmentary aggregates/melanomacrophage centres; PD, parenchymal disorganisation; Pv, portal vein; S, sinusoids; VC, vascular congestion; VF, vacuole formation.

Figure 3 shows H&E-stained liver sections before and after exposure. In photomicrographs A–E, prior to exposure, the hepatic parenchyma shows vacuolated hepatocytes with a homogeneous pattern and preserved cordonal/sinusoidal organisation. In the supplemented treatments, especially C–E, the tissue arrangement is more regular, with no apparent necrosis or marked congestion. Cytoplasmic vacuolisation appears physiological or metabolic and consistent with energy storage rather than significant degenerative injury.

In photomicrographs F–J, the exposed control (panel F) shows the most obvious damage after exposure: parenchymal disorganisation, greater cellular irregularity, areas of marked congestion/vascularisation, pigmentary aggregates and partial loss of the regular hepatocellular pattern. Alterations are still seen in G and H, but apparently with less severity than in F. Hepatic architecture appears to be better preserved in I and J with more organised hepatocytes, less disruption of the parenchyma, and no widespread severe lesions.

Table 7 shows that the hepatic histomorphology of *O. niloticus* was significantly affected by dietary OEO levels before and after malathion exposure (*p* < 0.05), except for nuclear area after exposure, where no significant differences were observed (*p* > 0.05). Before exposure, hepatocyte area, nuclear area, cytoplasmic area and the cytoplasm-to-nucleus ratio increased significantly with supplementation, with the highest values recorded in the 2.25 and 3.00 g kg^−1^ treatments. Hepatocyte area reached its maximum value at 3.00 g kg^−1^ (79.02 µm^2^), as did nuclear area (6.10 µm^2^) and cytoplasmic area (72.92 µm^2^), whereas the cytoplasm-to-nucleus ratio was highest at 2.25 g kg^−1^ (12.13).

After exposure, a different pattern was observed. Hepatocyte area, cytoplasmic area and the cytoplasm-to-nucleus ratio were significantly higher in the exposed control group, with values of 93.6 µm^2^, 87.14 µm^2^ and 13.51, respectively, and decreased in the supplemented treatments.

### 3.7. Histology of Kidney

**Figure 4:**
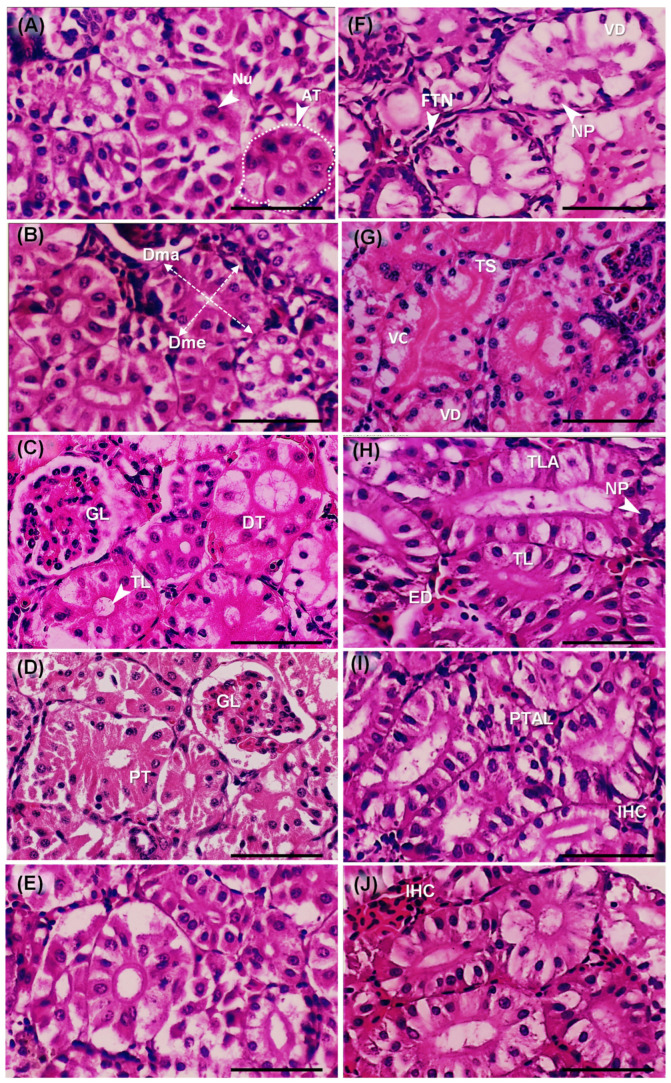
Kidney histological micrographs of *O. niloticus* fed diets supplemented with 0, 0.75, 1.50, 2.25 and 3.00 g kg^−1^ OEO for eight weeks before exposure (**A**–**E**) and after 96 h of malathion-induced stress under the same dietary treatments (**F**–**J**), respectively. Haematoxylin and eosin (H&E) staining. Scale bar: 20 µm. Total magnification: 400×. Abbreviations: Nu, nucleus; AT, altered tubule; Dma, major diameter; Dme, minor diameter; GL, glomerulus; GA, glomerular atrophy; PT, proximal tubule; DT, distal tubule; TL, tubular lumen; VD, vacuolar degeneration; VC, vascular congestion; TS, tubular swelling; FTN, focal tubular necrosis; NP, nuclear pyknosis; ED, epithelial desquamation; TLA, tubular lumen alteration; PTAL, partial tubular architecture loss; IHC, increased hematopoietic/interstitial cellularity.

Figure 4 shows H&E-stained kidney sections before and after exposure. Before the toxic challenge, supplemented fish showed a more organised renal architecture, consistent with improved filtration, reabsorption and osmotic regulation capacity. In contrast, after exposure, the control group showed a pattern compatible with acute toxic nephropathy, presumably associated with glomerular retraction, tubular swelling, luminal dilation, vacuolisation and epithelial disorganisation. The lower severity of these lesions in the supplemented groups suggests that OEO limited organophosphate-induced renal deterioration.

Table 8 shows that kidney histomorphology in *O. niloticus* was significantly affected by dietary levels of OEO before and after malathion exposure (*p* < 0.05). Before exposure, glomerular area, tubular area, luminal area, tubular wall area and the number of nuclei in distal and proximal tubules differed significantly among treatments. The highest values were recorded at 2.25 and 3.00 g kg^−1^. After exposure, glomerular area was lower in the control group and higher in the supplemented treatments. In contrast, tubular area, luminal area and tubular wall area in distal and proximal tubules were higher in the exposed control group and decreased with supplementation, whereas the number of nuclei per tubule increased in the OEO treatments, especially at 2.25 and 3.00 g kg^−1^.

### 3.8. Multivariate Patterns of Functional Response

**Figure 5:**
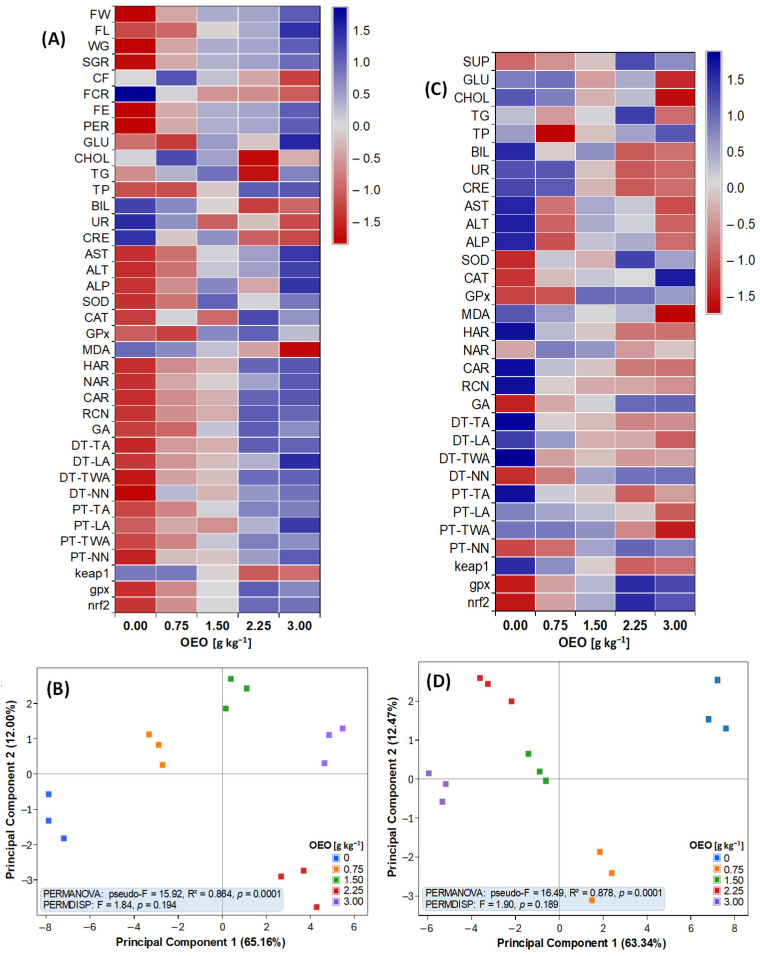
Multivariate patterns in *O. niloticus* fed diets supplemented with OEO for 8 weeks and subsequently exposed to malathion-induced stress for 96 h. (**A**) Heatmap analysis after the 8-week feeding period; (**B**) PCA analysis after the 8-week feeding period; (**C**) heatmap analysis after 96 h of malathion-induced stress; (**D**) PCA analysis after 96 h of malathion-induced stress. FW (Final body weight), FL (Final length), WG (Weight gain), SGR (Specific growth rate), CF (Condition factor), FCR (Feed conversion ratio), FE (Feed efficiency), PER (Protein efficiency ratio), GLU (Glucose), CHOL (Cholesterol), TG (Triglycerides), TP (Total protein), AST (Aspartate aminotransferase), ALT (Alanine aminotransferase), ALP (Alkaline phosphatase), SOD (Superoxide dismutase enzyme), CAT (Catalase enzyme), GPx (Glutathione peroxidase enzyme), MDA (Malondialdehyde), HAR (Hepatocyte area), NAR (Hepatocyte nucleus area), CAR (Hepatocyte cytoplasm area), RCN (Cytoplasm-to-nucleus area ratio), GA (Glomerular area), DT-TA (Distal tubular area), DT-LA (Distal tubular luminal area), DT-TWA (Distal tubular wall area), DT-NN (Number of nuclei in distal tubules), PT-TA (Proximal tubular area), PT-LA (Proximal tubular luminal area), PT-TWA (Proximal tubular wall area), PT-NN (Number of nuclei in proximal tubules), *nrf2* (Nuclear factor erythroid 2-related factor 2 gene), *gpx* (Glutathione peroxidase gene), and *keap1* (Kelch-like ECH-associated protein gene).

After 8 weeks of feeding (heatmap, Figure 5A), a clear dose-dependent pattern was observed: 0–0.75 g kg^−1^ showed mainly negative values for growth, feed-use efficiency, and antioxidant-related traits, whereas 2.25–3.00 g kg^−1^ displayed mainly positive values. FW increased from −1.74 to +1.10, FL from −1.13 to +1.46, WG from −1.74 to +1.10, and SGR from −1.66 to +0.98; FCR decreased from +1.84 to −0.96, with FE and PER rising from about −1.75 to +1.10. GLU and TP increased from −0.80 to +1.55 and from −1.09 to +1.14, whereas CAT and GPx peaked at 2.25 g kg^−1^ (+1.25 and +1.06) and MDA decreased from +1.01 to −1.69.

In the PCA after 8 weeks of feeding (Figure 5B), the first two components explained. 77.16% of the total variance, with PC1 accounting for 65.16% and PC2 for 12.00%. The control group (0 g kg^−1^) was clearly positioned on the negative side of PC1, whereas the 2.25 and 3.00 g kg^−1^ groups were located on the positive side, indicating marked multivariate separation between the unsupplemented and higher-supplemented fish. The PCA loadings showed that PC1 was mainly associated with growth and feed utilisation variables, metabolic biomarkers and tissue-related indicators. Positive loadings were represented principally by FW, WG, FE, PER and selected tissue-preservation indicators, whereas FCR, BIL, UR and MDA loaded negatively. The complete loading matrix is provided in the Supplementary Materials (Figure S2, Table S1). These differences were further supported by exploratory PERMANOVA (pseudo-F = 15.92, R^2^ = 0.864, *p* = 0.0001), while the non-significant PERMDISP result (F = 1.84, *p* = 0.194) indicated that the observed separation was mainly driven by differences in centroid location rather than dispersion.

Multivariate differences remained after malathion exposure (heatmap, Figure 5C), with a dose-dependent trend: 0–0.75 g kg^−1^ was mainly associated with positive values for stress- and damage-related indicators, while 2.25–3.00 g kg^−1^ was associated with positive values for antioxidant and protective traits. BIL, UR, CRE, AST, ALT and ALP were highest in the control (Z = +1.59, +1.19, +1.27, +1.62, +1.67 and +1.60) and decreased to negative values at 3.00 g kg^−1^. CHOL and MDA also decreased from +1.15 and +1.13 to −1.68 and −1.77 while CAT increased from −1.38 to +1.70 and *gpx*/*nrf2* shifted from −1.56/−1.60 to +0.82/+0.71.

After 96 h of malathion-induced stress (Figure 5D), the first two components explained 75.81% of the total variance, with PC1 accounting for 63.34% and PC2 for 12.47%. The control group remained on the positive side of PC1, whereas the 2.25 and 3.00 g kg^−1^ groups were mainly located on the negative side. The 1.50 g kg^−1^ treatment showed an intermediate position, whereas 0.75 g kg^−1^ was additionally distinguished by negative PC2 scores. PC1 was mainly associated with tissue-related indicators and redox gene expression markers, with strong positive loadings for HAR, CAR, *keap1*, DT-TA and PT-TA, and strong negative loadings for *gpx*, *nrf2*, GA and DT-NN (Figure S3, Table S2). PC2 was mainly influenced by TP, ALP, ALT, AST, NAR and TG. This pattern was likewise supported by exploratory PERMANOVA (pseudo-F = 16.49, R^2^ = 0.878, *p* = 0.0001), with a non-significant PERMDISP result (F = 1.90, *p* = 0.189), indicating that treatment differences were primarily associated with centroid separation rather than unequal within-group dispersion.

Together, the heatmap and PCA indicated that OEO was associated with multivariate response patterns in Nile tilapia at physiological and transcriptional levels, with higher inclusion levels, particularly 2.25 and 3.00 g kg^−1^, consistently separated from the control before and after malathion exposure, indicating a shift toward a more favourable integrated response.

## 4. Discussion

### 4.1. Phytochemical Profile of OEO

The chromatographic profile of OEO confirms that the oil used in this study corresponds to a carvacrol-dominant chemotype, which is consistent with profiles previously described for *O. vulgare*, particularly for materials rich in phenolic monoterpenes. In the present study, carvacrol was the major component, followed by γ-terpinene, *p*-cymene and (E)-β-caryophyllene, a hierarchy very close to that reported by Azizi et al. [39], who identified 42 compounds in three populations of *O. vulgare* and found carvacrol as the main constituent, reaching 77.4%, followed by γ-terpinene and *p*-cymene. Similarly, Stoilova et al. [66] described an OEO with 33 identified compounds, accounting for 98.8% of the total composition, and characterised by the predominance of carvacrol, *p*-cymene, γ-terpinene and β-caryophyllene, a pattern that supports the chemical consistency of the OEO evaluated in the present study. Milovanović et al. [41] also reported 33 volatile compounds in oregano essential oil with a clear carvacrol-dominant profile, thus supporting the occurrence of carvacrol-rich chemotypes within oregano essential oils.

Nevertheless, marked intraspecific variability has also been reported in *O. vulgare*, with chemotypes dominated by carvacrol, thymol or terpinen-4-ol, attributable to genetic origin, agroecological conditions, phenological stage and post-harvest processing; therefore, phytochemical characterisation is necessary to properly understand the biological response observed [67,68,69]. Functionally, the high proportion of carvacrol, together with thymol and the biosynthetically related precursors γ-terpinene and *p*-cymene, provides a plausible phytochemical basis for the antioxidant and cytoprotective effects attributed to OEO, considering the redox-modulating and antimicrobial properties described for phenolic monoterpenes [70,71].

### 4.2. Growth and Survival

Dietary supplementation with OEO consistently improved the zootechnical performance of *O. niloticus*, as reflected by increased growth and protein efficiency, together with a progressive reduction in feed conversion ratio. This improvement does not appear to be mainly explained by a palatability effect or by increased feed intake, since apparent feed intake remained within a narrow range among treatments. Rather, this pattern suggests that OEO favoured better feed utilisation and acted as a functional modulator of fish biological efficiency, beyond a simple sensory effect.

This response may be related to the complex phytochemical matrix of OEO, characterised by the presence of phenolic monoterpenes, monoterpene hydrocarbons and oxygenated terpenoids. Although compounds such as carvacrol, γ-terpinene and *p*-cymene have previously been associated with improved digestive and intestinal functionality, lower oxidative pressure and reduced physiological costs under stress, the results of the present study should be interpreted as a response to the whole OEO rather than to individual constituents, since isolated compounds or purified fractions were not evaluated [29,33]. In Nile tilapia, OEO supplementation has been reported to improve growth, feed efficiency and intestinal integrity, particularly under stressful conditions [35], supporting the productive results observed in the present study, probably by promoting better digestive utilisation and a more stable physiological condition. Similarly, in *Ictalurus punctatus*, OEO improved growth [27], suggesting that the phytochemical matrix of oregano may promote responses compatible with greater anabolic efficiency and adaptive capacity of the host.

The 100% survival recorded in all groups during the eight weeks before the challenge supports the dietary safety of OEO at the tested doses. However, after malathion exposure, survival was higher in fish supplemented with 2.25 and 3.00 g kg^−1^ OEO than in the control group, indicating that its effect was also associated with greater physiological robustness against the toxic stressor. Consequently, supplemented fish probably faced the challenge under better functional conditions, with greater capacity to maintain homeostasis and attenuate the metabolic cost of intoxication [29,72]. These results indicate that OEO improved the biological efficiency and robustness of *O. niloticus*.

### 4.3. Metabolic Biomarkers

Metabolic biomarkers indicate that OEO modulated the basal physiological status and the response to malathion exposure [73,74]. Before exposure, the increase in GLU and TP, together with the reduction in BIL, UREA and CREA in the 2.25–3.00 g kg^−1^ treatments, suggests greater availability of energetic substrates, a possible improvement in hepatic biosynthetic capacity and a lower hepatorenal burden [75]. In this context, the simultaneous improvement in zootechnical parameters, particularly greater growth, lower feed conversion ratio and higher protein efficiency, suggests that the basal metabolic changes formed part of an integrated physiological response oriented towards more efficient nutrient utilisation.

Although AST, ALT and ALP are commonly associated with hepatocellular or hepatobiliary alteration, their basal increase in fish supplemented with OEO occurred within a broader physiological context, characterised by better productive performance, lower BIL, UREA and CREA levels, lower lipid peroxidation, higher antioxidant activity and absence of evident histological deterioration before exposure. In this scenario, these enzymatic variations could form part of a basal metabolic and hepatobiliary modulation associated with a greater demand for nutrient processing, without excluding that they may also reflect an adaptive hepatic response to dietary OEO inclusion. This is consistent with previous studies in which OEO improved intestinal morphometry and hepatorenal function in *Cyprinus carpio*, whereas in *Dicentrarchus labrax*, favourable modulation of metabolic status has been described [28,36]. These findings support the physiological plausibility that OEO may favour better digestive and metabolic utilisation, with positive effects on protein synthesis and somatic growth [29,36].

After malathion exposure, the 2.25 and 3.00 g kg^−1^ OEO treatments attenuated the metabolic and hepatorenal alterations observed in the control group, suggesting a lower physiological cost associated with intoxication. The hyperglycaemia recorded in the control group is consistent with a neuroendocrine stress response, involving intensified glycogenolysis and gluconeogenesis to sustain detoxification and tissue repair [73,76]. In parallel, the increase in UREA and CREA is compatible with altered renal function and lower efficiency in the elimination of nitrogenous metabolites, whereas the elevation of BIL, AST, ALT and ALP suggests possible hepatobiliary alteration, increased membrane permeability and enzymatic leakage associated with cellular damage [10,75].

By contrast, the post-challenge decrease in CHOL and the maintenance of TP, particularly in fish fed 3.00 g kg^−1^ OEO, suggest that supplementation helped to preserve hepatic homeostasis and biosynthetic capacity, attenuating malathion-induced hepatorenal dysfunction and metabolic imbalance [76]. The effects of OEO on metabolic biomarkers support a more stable systemic response under chemical stress, in agreement with improved productive performance and greater functional preservation of the liver and kidney [10,29].

### 4.4. Antioxidant Capacity

The antioxidant response indicates that dietary supplementation with OEO was associated with improved regulation of redox status in *O. niloticus*, both under basal conditions and after malathion-induced stress [16,77]. Before exposure, the increased activity of the antioxidant enzymes superoxide dismutase, catalase and glutathione peroxidase, together with the progressive decrease in MDA towards the 2.25–3.00 g kg^−1^ treatments, suggests greater basal antioxidant capacity and lower susceptibility of cell membranes to lipid peroxidation [16]. This pattern is compatible with the combined action of the complex phytochemical matrix of OEO, composed of phenolic monoterpenes, monoterpene hydrocarbons and oxygenated terpenoids, which have previously been associated with modulation of oxidative status in fish [33,78,79]. OEO has consistently been reported to improve antioxidant status in Nile tilapia under stressful conditions and to increase antioxidant capacity and resistance to biological challenges in *Ictalurus punctatus*. Similarly, favourable hepatorenal responses have been described in *Cyprinus carpio* and *Dicentrarchus labrax* after supplementation with *Origanum vulgare*, supporting the plausibility of a protective effect that may depend on the physiological context, dose and experimental model [27,29,36,76,80].

Malathion exposure modified the antioxidant response in all groups; however, supplemented fish showed higher activity of the enzymes superoxide dismutase, catalase and glutathione peroxidase, together with lower MDA concentrations than the control group, especially in the 2.25–3.00 g kg^−1^ treatments [12,16]. This behaviour suggests that OEO was associated with a greater capacity to contain organophosphate-induced overproduction of reactive oxygen species [78,79]. The increase in MDA in the exposed control group is consistent with greater oxidation of membrane lipids, whereas its decrease in the supplemented groups points to more effective containment of secondary peroxidation [80,81].

The differential response of antioxidant enzymes provides functional information on redox balance. The higher activity of the enzyme superoxide dismutase observed in fish supplemented with 2.25 g kg^−1^ suggests a greater capacity to neutralise superoxide, whereas the higher activity of the enzyme catalase in the 3.00 g kg^−1^ treatment indicates a more intense response against hydrogen peroxide. Together with glutathione peroxidase enzyme activity, this pattern suggests better control of hydroperoxides and lower overall peroxidative pressure [34]. OEO was associated with a more efficient enzymatic antioxidant response and reduced malathion-induced lipid peroxidation, which is compatible with greater preservation of membrane integrity under chemical stress [76,78].

### 4.5. Antioxidant Gene Expression

The relative expression of *nrf2*, *keap1* and *gpx* indicates that dietary supplementation with OEO was associated with transcriptional modulation of genes linked to redox regulation, in agreement with the functional antioxidant response described previously [21,22]. Before exposure, the progressive increase in *nrf2* and *gpx*, together with the decrease in *keap1* towards the 2.25–3.00 g kg^−1^ treatments, suggests a basal adjustment of antioxidant regulation at the transcriptional level [24,64]. This pattern is compatible with a lower repressive signal associated with *keap1* and with higher expression of markers related to the antioxidant response, such as *nrf2* and *gpx* [19,64]. Within this transcriptional framework, the coordinated increase in *nrf2* and *gpx*, together with the reduction in *keap1*, suggests a more favourable redox profile in fish receiving the highest OEO levels. This transcriptional profile agrees with the higher activities of antioxidant enzymes and the lower lipid peroxidation described previously, reinforcing the interpretation of a more coordinated redox response in supplemented fish [82,83].

After exposure, the control group showed the lowest expression of *nrf2* and *gpx* and the highest expression of *keap1*, configuring a less favourable transcriptional profile under malathion-induced oxidative stress. This pattern suggests that, under organophosphate pressure, the endogenous antioxidant response of the control group was insufficient to compensate for the increase in oxidative stress [13,78]. In this scenario, lower *gpx* expression could limit the response capacity against hydroperoxides, in agreement with the higher MDA concentration and histological deterioration observed in the control group [11,82]. Conversely, fish fed 2.25 and 3.00 g kg^−1^ OEO showed higher relative expression of *nrf2* and *gpx*, together with lower *keap1* values, suggesting a more favourable transcriptional modulation against the malathion challenge [22,24]. The higher expression of *gpx* is particularly relevant, since the functional product of this gene, the glutathione peroxidase enzyme, participates in the reduction of lipid hydroperoxides and may contribute to limiting the propagation of oxidative damage in membrane phospholipids. This is consistent with the lower MDA concentrations and greater tissue preservation observed in the supplemented groups [82]. In Nile tilapia and other fish, more favourable regulation of genes associated with the Keap1–Nrf2 pathway has been linked to lower inflammation and apoptosis, as well as better structural preservation under stress conditions [21,24,63]. Therefore, the modulation of *nrf2*, *keap1* and *gpx* observed in the present study supports the interpretation that OEO was associated with a more coordinated antioxidant and transcriptional response against malathion-induced stress, compatible with the lower oxidative and histological damage observed after exposure.

### 4.6. Liver Histology

Liver histology showed that OEO supplementation modulated the structural organisation of the liver of *O. niloticus* according to physiological status, favouring basal functionality and limiting hepatocellular injury under malathion-induced stress [84,85]. Before exposure, the increase in HAR, NAR, CAR, and RCN at 2.25–3.00 g kg^−1^ indicates a hepatocyte configuration compatible with greater biosynthetic and metabolic activity. In teleosts, larger hepatocyte volume accompanied by an increased cytoplasm:nucleus ratio is usually associated with more favourable hepatic function [28,36]. Accordingly, supplemented groups showed more voluminous hepatocytes, relatively homogeneous cytoplasm, defined nuclei, preserved cord-like arrangement, patent sinusoids, and little evidence of inflammatory infiltrate. This pattern is consistent with that reported in hybrid tilapia supplemented with *Tithonia diversifolia*, where preserved hepatic architecture, continuous hepatocyte cords and plates, defined nuclei, and absence of severe pathological changes were observed [50].

The morphometric increase in the control group after exposure to malathion should be due to hepatocellular expansion caused by injury rather than functional hypertrophy. In fish, rapid increases in hepatocyte and cytoplasmic area following exposure to toxicants are usually linked to cellular swelling, hydropic degeneration, vacuolisation, and sinusoidal congestion particularly when coupled with increases in AST, ALT and MDA [10,12].

In the present study, the exposed control group showed pyknosis, focal necrosis, and inflammatory infiltrate, configuring hepatocellular injury associated with redox imbalance and membrane alteration. Méndez-Martínez et al. [49] described focal necrosis, cytoplasmic vacuolisation and sinusoidal congestion in the unprotected challenged group in *Dormitator latifrons*, while supplemented treatments better maintained hepatic organisation. In contrast, the lower morphometric expansion in the OEO-supplemented groups after malathion exposure indicates attenuation of organophosphate-induced hepatocellular injury [33,86]. This preservation was correlated with less severe vacuolisation, better preservation of the cord-like arrangement, less inflammatory infiltrate and greater nuclear integrity, suggesting a more effective containment of tissue damage [84]. Moreover, this result was accompanied by a lower change in AST, ALT and MDA in supplemented groups, which reinforces the fact that the hepatic protection was the structural expression of a higher metabolic and redox stability [12,86].

### 4.7. Kidney Histology

In teleosts, the kidney performs osmoregulatory, excretory, and immunohaematopoietic functions; therefore, glomerular and tubular morphometry reflects filtration, reabsorption, and hydroelectrolytic homeostasis, with better renal organisation indicating greater functional stability [87,88,89]. Before exposure, the increase in glomerular area, larger tubular dimensions, and higher number of nuclei, particularly at 2.25 and 3.00 g kg^−1^ OEO, suggest greater renal functional capacity. This pattern may be explained by improved metabolic and redox status, which favours glomerular perfusion, preserves tubular epithelium, and supports ATP-dependent ion transporters [88]. Accordingly, supplemented fish showed well-delimited glomeruli, intact tubular epithelium, regular lumina, and preserved nuclei, indicating stable tissue organisation. This response agrees with studies in fish supplemented with functional compounds that preserve tissue integrity and hepatorenal function [28,33,90].

The decrease in glomerular area observed in the control group following malathion exposure, in conjunction with tubular and luminal enlargement, suggests impaired glomerular filtration and epithelial integrity. This pattern is in line with organophosphate-induced ROS generation, lipid peroxidation and mitochondrial dysfunction, leading to decreased ATP availability and compromised ion transporters [79]. In the renal epithelium, these changes result in impairment of the osmotic and volume regulation, leading to cellular swelling, vacuolisation, luminal expansion and epithelial disorganisation [91,92]. Simultaneously, glomerular retraction can cause a decrease in effective filtration area and impact nitrogenous metabolite clearance [10]. The control group with a higher level of UREA and CREA supports functional renal alteration consistently [73,93]. Histologically, the exposed fish showed retracted glomeruli, dilated tubules, disorganised epithelium and reduced nuclear preservation, configuring glomerulotubular injury associated with oxidative stress and renal impairment [91].

However, the presence of glomerular preservation, less tubular dilation and the increased number of nuclei in supplemented groups indicated that OEO attenuated malathion-induced kidney injury. This protection may be due to better antioxidant defence, limiting membrane peroxidation, preserving mitochondrial function, reducing ionic leakage and cellular oedema, thus reducing luminal expansion and epithelial disruption [33,90]. Furthermore, the increased number of nuclei per tubule indicates preservation of the renal epithelium and ability for tubular reabsorption and secretion. This improved renal profile coincided with lower UREA, CREA and MDA levels, as well as favourable modulation of genes related to the Keap1–Nrf2 pathway and *gpx* expression, which has been associated with lower inflammation, apoptosis and histological damage in tilapia [22,64,82].

### 4.8. Multivariate Patterns of Functional Response

The multivariate analysis integrated productive performance, metabolic and antioxidant status, gene expression, and hepatic and renal histomorphology in *O. niloticus* fed OEO under basal conditions and malathion-induced stress. Before exposure, the association of the highest OEO doses with growth, feed efficiency, antioxidant capacity, and tissue-integrity traits suggests a more efficient physiological organisation, in which nutrient utilisation, redox stability, and hepatorenal condition converged. This pattern agrees with studies in tilapia reporting simultaneous improvements in growth, feed utilisation, and antioxidant status after dietary *Origanum vulgare* supplementation [29,33]. Therefore, the clustering of FW, WG, SGR, PER and FCR together with SOD, CAT, GPx and protective histological traits supports the interpretation that OEO promoted a basal profile consistent with a higher metabolic efficiency, a better antioxidant control and a higher tissue integrity.

Post malathion exposure, the multivariate configuration showed a clear separation between the control and supplemented groups indicating a shift from a phenotype characterised by oxidative modification, metabolic imbalance and hepatorenal damage to a more protective condition at the highest OEO doses. In the control-associated pole, variables related to toxicity, including MDA, UREA, CREA, BIL, and traits compatible with hepatocellular and glomerulo tubular injury, clustered. On the other hand, the supplemented groups focused on CAT, *gpx*, *nrf2*, hepatic and renal preservation and a more favourable productive profile. This reorganisation is consistent with organophosphate toxicity which is mostly accompanied by overproduction of reactive oxygen species, lipid peroxidation, membrane damage and metabolic disruption [78].

The combined association of CAT, *gpx*, *nrf2* and protective tissue traits suggests that OEO supplementation was associated with reduced oxidative stress and a more favourable transcriptional and tissue-preservation profile. Therefore, the improvements in growth, metabolic homeostasis, antioxidant capacity, and histological preservation were not isolated responses, but interdependent components of a physiological resilience phenotype. This interpretation agrees with evidence from fish exposed to pesticides and with studies showing that *O. vulgare* can mitigate toxicant-induced oxidative, biochemical, and histopathological alterations [33,94]. Thus, as an integrative inference from exploratory patterns, OEO appears to shift *O. niloticus* from oxidative–metabolic imbalance towards a more stable physiological condition, supporting *nrf2*-linked antioxidant and metabolic modulation under organophosphate-induced stress.

## 5. Conclusions

Dietary supplementation with oregano essential oil (OEO) was associated with improved productive performance, feed utilisation and post-challenge survival in Nile tilapia subjected to an acute malathion challenge based on the 96 h median lethal concentration. Fish supplemented with OEO, particularly at 2.25 and 3.00 g kg^−1^, showed a more favourable integrated physiological profile, characterised by lower lipid peroxidation, higher antioxidant enzyme activity, attenuated post-exposure biochemical alterations, and better preservation of liver and kidney structure. At the transcriptional level, supplemented fish showed higher relative expression of *nrf2* and *gpx*, together with lower *keap1* expression, suggesting that OEO supplementation was associated with modulation of Keap1–Nrf2-related transcriptional responses and gpx expression, in agreement with the antioxidant and histological profiles observed.

The multivariate patterns supported an integrated separation between the exposed control and the groups supplemented with the highest OEO doses, consistent with a coordinated antioxidant, metabolic and tissue-preservation response. Overall, the results indicate that OEO is a promising phytogenic additive for Nile tilapia nutrition, with potential to improve physiological resilience against severe acute organophosphate stress. Future studies should validate these responses under chronic and environmentally relevant exposure scenarios and further clarify the specific contribution of individual OEO constituents.

## Figures and Tables

**Table 1:** Formulations and chemical composition of diets with the inclusion of OEO at different levels.

Ingredients (g kg^−1^)	OEO Levels (g kg^−1^)				
	0 (Control)	0.75	1.50	2.25	3.00
Fishmeal ^1^	256.50	256.50	256.50	256.50	256.50
Soybean meal ^2^	261.50	261.50	261.50	261.50	261.50
Wheat flour ^3^	217.50	217.50	217.50	217.50	217.50
Maize flour ^4^	209.80	209.05	208.30	207.55	206.80
OEO	0.00	0.750	1.500	2.250	3.000
Fish oil ^5^	15.00	15.00	15.00	15.00	15.00
Unflavoured gelatine ^6^	2.50	2.50	2.50	2.50	2.50
Proteases ^7^	0.50	0.50	0.50	0.50	0.50
Monocalcium phosphate ^8^	0.50	0.50	0.50	0.50	0.50
Mineral premix ^9†^	15.00	15.00	15.00	15.00	15.00
Vitamin premix ^10‡^	20.00	20.00	20.00	20.00	20.00
Ascorbic acid ^11^	1.00	1.00	1.00	1.00	1.00
BHT ^12^	0.20	0.20	0.20	0.20	0.20
Chemical composition (g kg^−1^, dry matter basis) *					
Crude protein	343.64	343.57	343.5	343.44	343.37
Ether extract	54.43	55.15	55.86	56.58	57.29
Ash	102.49	102.48	102.46	102.45	102.44
Crude fibre	21.35	21.34	21.32	21.31	21.29
NFE	478.09	477.47	476.85	476.23	475.6
Gross energy (MJ kg^−1^)	16.63	16.65	16.66	16.68	16.69
Digestible energy (MJ kg^−1^)	14.82	14.83	14.85	14.86	14.88

* Data are reported as the mean of three replicates. Ingredients were purchased from the following suppliers: ^1,2,3,4^ Agripac, located in Santo Domingo de los Tsáchilas, Coop. Higuilpe neighbourhood and Av. de los Colonos, Block T; ^5,6,7,8,9,10,11,12^ Química La Fórmula, June Guzmán, Quevedo, Ecuador. ^†^ The premix contained the following, mg kg^−1^: magnesium sulphate 5.1; sodium chloride 2.4; potassium chloride 2; ferrous sulphate 1; z inc sulphate 0.2; manganese sulphate 0.0314; sulphate of manganese 0.1015; cobalt sulphate 0.0191; calcium iodate 0.0118; and choline chloride 0.051. ^‡^ This premix contained the following, mg kg ^−1^: thiamine 60; riboflavin 25; niacin 40; vitamin B6 50; pantothenic acid 75; biotin 1; folate 10; vitamin B12 0.2; vitamin K5 0.1; choline 600; myoinositol 400; vitamin C 200; vitamin A 5000 IU; vitamin E 100; and vitamin D 0.1. Abbreviation: NFE, nitrogen-free extract.

**Table 2:** Primer sequences used for gene expression analysis.

Gene	Primer Sequence (5′–3′)	Accession No.	Product Size (bp)	Annealing Temperature (°C)	Efficiency (%)	R^2^
*nrf2*	F: CTGCCGTAAACGCAAGATGG R: ATCCGTTGACTGCTGAAGGG	XM_003447296.4/.5	226	57	96.8	0.993
*keap1*	F: CTTCGCCATCATGAACGAGC R: CACCAACTCCATACCGCACT	XM_003447926.3/.4	181	57	94.7	0.991
*gpx*	F: GGAACGACAACCAGGGACTA R: TCCCTGGACGGACATACTTC	GQ853451.1	160	60	98.5	0.995
*18S rRNA*	F: GGACACGGAAAGGATTGACAG R: GTTCGTTATCGGAATTAACCAGAC	JF698683	111	65	101.2	0.997
*ef1α*	F: GCACGCTCTGCTGGCCTTT R: GCGCTCAATCTTCCATCCC	AB075952	250	66	97.6	0.994

*Gene: nrf2*, nuclear factor erythroid 2-related factor 2; *keap1*, Kelch-like ECH-associated protein 1; *gpx*, glutathione peroxidase; 18S rRNA, 18S ribosomal RNA; *ef1α*, elongation factor 1-alpha. Primer sequences, accession numbers, product sizes, and annealing temperatures were obtained from previous studies [62,63,64,65]. Amplification efficiency, and R^2^ were determined in the present study using serial dilutions of pooled cDNA.

**Table 3:** Phytochemical compounds identified in OEO.

	Compound	RT, Min	RI Exp.	Po, %	Chemical Class
1	α-Thujene	5.53	926	0.2	Mh
2	α-Pinene	7.08	932	0.4	Mh
3	Camphene	7.42	948	0.1	Mh
4	Sabinene	8.03	973	0.3	Mh
5	β-Pinene	8.57	977	0.6	Mh
6	1-Octen-3-ol	9.43	980	0.1	Ava
7	Myrcene	9.87	989	1.1	Mh
8	α-Phellandrene	10.08	1005	0.8	Mh
9	Δ-3-Carene	10.57	1010	0.9	Mh
10	α-Terpinene	10.93	1016	4.3	Mh
11	*p*-Cymene	11.36	1023	6.9	Mh
12	Limonene	11.78	1030	1.0	Mh
13	β-Phellandrene	12.03	1031	0.3	Mh
14	(Z)-β-Ocimene	12.52	1037	0.7	Mh
15	γ-Terpinene	13.34	1058	9.0	Mh
16	cis-Sabinene hydrate	13.87	1072	0.7	Om
17	Terpinolene	14.92	1084	0.3	Mh
18	Linalool	15.74	1096	1.1	Om
19	trans-Sabinene hydrate	16.63	1103	0.5	Om
20	Borneol	17.47	1171	0.3	Om
21	Terpinen-4-ol	18.32	1183	0.5	Om
22	α-Terpineol	19.53	1195	0.4	Om
23	Carvacrol methyl ether	20.13	1241	0.2	Pmd
24	Thymoquinone	20.98	1250	0.5	Oam
25	Thymol	21.88	1291	0.5	Pm
26	Carvacrol	22.67	1305	61.6	Pm
27	Carvacryl acetate	24.07	1363	0.6	Pmd
28	(E)-β-Caryophyllene	25.33	1418	5.1	Sm
29	α-Humulene	26.18	1457	0.3	Sm
30	Caryophyllene oxide	27.08	1586	0.4	Os
31	epi-α-Muurolol	30.12	1642	0.2	Os
32	α-Eudesmol	31.57	1662	0.1	Os

Abbreviations: RT, retention time; RI exp., experimental retention index; Po, percentage obtained by peak-area normalisation, Mh, monoterpene hydrocarbon; Ava, aliphatic volatile alcohol; Om, oxygenated monoterpene; Pm, phenolic monoterpene; Pmd, phenolic monoterpene derivative; Oam, oxygenated aromatic monoterpenoid; Sm, sesquiterpene hydrocarbon; Os, oxygenated sesquiterpene.

**Table 4:** Growth performance in *O. niloticus* fed diets supplemented with OEO for 8 weeks and subsequently exposed to malathion-induced stress for 96 h.

Parameters	OEO Levels (g kg^−1^)					*p*-Value
	0 (Control)	0.75	1.50	2.25	3.00	
Before exposure						
Initial weight, g	5.79 ± 0.02 ^a^	5.72 ± 0.09 ^a^	5.77 ± 0.08 ^a^	5.72 ± 0.12 ^a^	5.79 ± 0.08 ^a^	0.8886
Final weight, g	20.76 ± 0.11 ^d^	24.33 ± 0.08 ^c^	26.40 ± 0.11 ^b^	26.52 ± 0.18 ^b^	28.02 ± 0.29 ^a^	0.0001
Final length, mm	113.41 ± 0.21 ^c^	115.28 ± 2.01 ^c^	121.90 ± 0.72 ^b^	125.09 ± 0.08 ^b^	132.11 ± 0.07 ^a^	0.0032
Weight gain, g	15.17 ± 0.13 ^d^	18.61 ± 0.05 ^c^	20.63 ± 0.19 ^b^	20.08 ± 0.09 ^b^	22.23 ± 0.31 ^a^	0.0001
SGR, % day^−1^	2.34 ± 0.02 ^c^	2.59 ± 0.02 ^b^	2.72 ± 0.02 ^ab^	2.74 ± 0.03 ^a^	2.82 ± 0.03 ^a^	0.0002
AFI (g fish^−1^day^−1^)	0.59 ± 0.01	0.59 ± 0.03	0.59 ± 0.02	0.60 ± 0.04	0.60 ± 0.02	0.0721
Condition factor	1.42 ± 0.01	1.61 ± 0.09	1.46 ± 0.09	1.36 ± 0.03	1.22 ± 0.02	0.0561
Feed conversion ratio	2.18 ± 0.02 ^d^	1.78 ± 0.01 ^c^	1.62 ± 0.04 ^b^	1.61 ± 0.01 ^b^	1.51 ± 0.03 ^a^	0.0012
Protein efficiency ratio	1.34 ± 0.01 ^d^	1.64 ± 0.01 ^c^	1.80 ± 0.02 ^b^	1.81 ± 0.03 ^b^	1.93 ± 0.03 ^a^	0.0011
Survival rate, %	100	100	100	100	100	ns
After exposure						
Survival rate, %	53.33 ± 1.92 ^d^	60 ± 0.51 ^c^	66.67 ± 0.50 ^b^	70 ± 3.33 ^ab^	76.67 ± 1.92 ^a^	0.0013

Results are reported as means ± standard error of 3 groups per treatment (*n* = 3). Different letters (abcd) indicate significant differences (*p* < 0.05). Abbreviations: SGR (Specific growth rate), AFI (Apparent feed intake), ns (not significant).

**Table 5:** Metabolic biomarkers in *O. niloticus* fed diets supplemented with *OEO* for 8 weeks and subsequently exposed to malathion-induced stress for 96 h.

Parameters	OEO Levels (g kg^−1^)					*p*-Value
	0 (Control)	0.75	1.50	2.25	3	
Before exposure						
Glucose, mg dL^−1^	34.02 ± 0.33 ^d^	29.04 ± 0.31 ^e^	49.05 ± 0.17 ^b^	42.03 ± 0.32 ^c^	60.01 ± 0.34 ^a^	0.0001
Cholesterol, mg dL^−1^	137.04 ± 0.84 ^dc^	152.02 ± 0.83 ^a^	143.03 ± 0.32 ^b^	112.05 ± 0.05 ^d^	131.07 ± 2.17 ^c^	0.0010
Triglycerides, mg dL^−1^	111.03 ± 0.07 ^b^	117.04 ± 0.06 ^ab^	121.03 ± 0.34 ^a^	104.05 ± 1.05 ^c^	120.02 ± 0.33 ^a^	0.0210
Total Protein, g dL^−1^	2.04 ± 0.01 ^c^	2.39 ± 0.02 ^c^	2.99 ± 0.03 ^b^	3.66 ± 0.07 ^a^	3.69 ± 0.01 ^a^	0.0001
Bilirubin, mg dL^−1^	0.42 ± 0.01 ^a^	0.39 ± 0.01 ^a^	0.35 ± 0.02 ^ab^	0.28 ± 0.01 ^b^	0.31 ± 0.02 ^ab^	0.0001
Urea, mg dL^−1^	7.2 ± 0.06 ^a^	6.55 ± 0.06 ^ab^	5.26 ± 0.05 ^c^	5.9 ± 0.05 ^bc^	5.1 ± 0.04 ^c^	0.0150
Creatinine, mg dL^−1^	0.82 ± 0.01 ^a^	0.66 ± 0.01 ^b^	0.74 ± 0.01 ^ab^	0.57 ± 0.01 ^c^	0.55 ± 0.01 ^c^	0.0050
AST, U L^−1^	11.45 ±0.17 ^c^	13.48 ±0.17 ^c^	17.54 ±0.36 ^b^	18.55 ± 0.50 ^b^	21.49 ± 0.21 ^a^	0.0011
ALT, U L^−1^	10.45± 0.20 ^c^	12.52 ±0.19 ^c^	16.00 ±0.53 ^b^	17.00± 0.67 ^ab^	19.50 ±0.17 ^a^	0.0001
ALP, U L^−1^	44.55 ± 0.17 ^c^	48.09± 0.33 ^b^	54.05± 0.42 ª	49.08± 0.62 ^b^	57.07± 0.33 ª	0.0001
After exposure						
Glucose, mg dL^−1^	103.02 ± 0.16 ^a^	104.01 ± 1.67 ^a^	97.05 ± 0.18 ^bc^	101.04 ± 0.05 ^ab^	93.06 ± 0.06 ^c^	0.0017
Cholesterol, mg dL^−1^	151.01 ± 0.33 ^a^	146.05 ± 0.05 ^b^	132.04 ± 0.83 ^d^	139.06 ± 0.17 ^c^	117.03 ± 0.83 ^e^	0.0001
Triglycerides, mg dL^−1^	111.05 ± 0.05 ^ab^	104.01 ± 1.33 ^ab^	109.04 ± 1.05 ^ab^	123.03 ± 5.17 ^a^	100.05 ± 0.16 ^b^	0.0304
Total Protein, g dL^−1^	2.78 ± 0.01 ^b^	2.01 ± 0.03 ^d^	2.55 ± 0.04 ^c^	2.76 ± 0.02 ^b^	2.97 ± 0.01 ^a^	0.0010
Bilirubin, mg dL^−1^	0.92 ± 0.01 ^a^	0.63 ± 0.01 ^ab^	0.76 ± 0.01 ^a^	0.49 ± 0.01 ^c^	0.52 ± 0.01 ^bc^	0.0202
Urea, mg dL^−1^	12.27 ± 0.12 ^a^	12.40 ± 0.30 ^a^	8.95 ± 0.09 ^b^	7.10 ± 0.07 ^c^	7.35 ± 0.07 ^c^	0.0031
Creatinine, mg dL^−1^	1.48 ± 0.02 ^a^	1.42 ± 0.10 ^a^	0.96 ± 0.05 ^b^	0.74 ± 0.01 ^c^	0.79 ± 0.06 ^c^	0.0010
AST, U L^−1^	20.45 ± 0.17 ^a^	12.50 ± 0.16 ^d^	16.53 ± 0.20 ^b^	15.47 ± 0.18 ^c^	11.52 ± 0.15 ^e^	0.0001
ALT, U L^−1^	18.54 ± 0.15 ^a^	10.46 ± 0.17 ^c^	14.48 ± 0.19 ^b^	13.52 ± 0.16 ^b^	10.55 ± 0.20 ^c^	0.0012
ALP, U L^−1^	56.05 ± 0.25 ^a^	43.06 ± 0.33 ^c^	49.09 ± 0.22 ^b^	50.07 ± 0.35 ^b^	44.04 ± 0.26 ^c^	0.0001

Results are reported as means ± standard error of 3 groups per treatment (*n* = 3). Different letters (abcde) indicate significant differences (*p* < 0.05). AST, Aspartate aminotransferase; ALT, Alanine aminotransferase; ALP, Alkaline phosphatase.

**Table 6:** Antioxidant capacity of *O. niloticus* fed diets supplemented with OEO for 8 weeks and subsequently exposed to malathion-induced stress for 96 h.

Parameters	OEO Levels (g kg^−1^)					*p*-Value
	0 (Control)	0.75	1.50	2.25	3.00	
Before exposure						
SOD, U mL^−1^	95.37 ± 1.55 ^c^	98.57 ± 0.44 ^bc^	108.57 ± 0.48 ^a^	103.68 ± 0.51 ^ab^	107.62 ± 0.83 ^a^	0.0004
CAT, U mL^−1^	47.76 ± 0.63 ^c^	53.66 ± 0.29 ^b^	49.43 ± 0.55 ^c^	58.22 ± 0.85 ^a^	55.82 ± 0.68 ^ab^	0.0002
GPx, U mL^−1^	92.29 ± 0.98 ^b^	90.79 ± 1.14 ^b^	102.84 ± 1.16 ^a^	104.79 ± 1.18 ^a^	100.29 ± 0.64 ^a^	0.0007
MDA, nmol mL^−1^	17.36 ± 0.20 ^a^	16.56 ± 0.30 ^ab^	15.23 ± 0.40 ^b^	13.25 ± 0.23 ^c^	9.32 ± 0.36 ^d^	0.0001
After exposure						
SOD, U mL^−1^	136.65 ± 1.17 ^c^	147.18 ± 0.718 ^b^	143.73 ± 0.92 ^b^	155.48 ± 0.67 ^a^	149.50 ± 1.60 ^b^	0.0014
CAT, U mL^−1^	62.24 ± 0.38 ^d^	69.00 ± 0.41 ^c^	71.57 ± 0.27 ^b^	70.38 ± 0.47 ^bc^	81.83 ± 0.31 ^a^	0.0001
GPx, U mL^−1^	139.21 ± 0.70 ^c^	140.31 ± 0.01 ^c^	158.98 ± 0.03 ^a^	158.70 ± 1.00 ^a^	154.90 ± 0.59 ^b^	0.0003
MDA, nmol mL^−1^	21.35 ± 0.03 ^a^	19.40 ± 0.32 ^b^	17.88 ± 0.15 ^b^	18.74 ± 0.24 ^b^	12.88 ± 0.52 ^c^	0.0001

Results are reported as means ± standard error of 3 groups per treatment (*n* = 3). Different letters (abcd) indicate significant differences (*p* < 0.05). Abbreviations: SOD (Superoxide Dismutase), CAT (Catalase), GPx (Glutathione Peroxidase), MDA (Malondialdehyde).

**Table 7:** Histomorphopathology of liver in *O. niloticus* fed diets supplemented with OEO for 8 weeks and subsequently exposed to malathion-induced stress for 96 h.

Parameters	OEO Levels (g kg^−1^)					*p*-Value
	0 (Control)	0.75	1.50	2.25	3.00	
Before exposure						
HAR, um^2^	46.53 ± 0.49 ^c^	57.08 ± 0.53 ^bc^	61.02 ± 0.17 ^b^	77.59 ± 2.09 ^a^	79.02 ± 2.24 ^a^	0.0001
NAR, um^2^	5.29 ± 0.03 ^c^	5.62 ± 0.07 ^bc^	5.73 ± 0.05 ^ab^	5.91 ± 0.01 ^ab^	6.10 ± 0.14 ª	0.0110
CAR, um^2^	41.24 ± 0.52 ^c^	51.46 ± 0.58 ^bc^	55.28 ± 0.21 ^b^	71.68 ± 2.09 ^a^	72.92 ± 2.18 ª	0.0001
RCN, um^2^	7.81 ± 0.14 ^c^	9.18 ± 0.20 ^bc^	9.65 ± 0.12 ^b^	12.13 ± 0.36 ^a^	11.97 ± 0.36 ª	0.0002
After exposure						
HAR, um^2^	93.6 ± 0.09 ^a^	85.71 ± 0.07 ^b^	83.6 ± 0.09 ^c^	80.59 ± 0.16 ^d^	80.45 ± 0.07 ^d^	0.0001
NAR, um^2^	7.47 ± 0.13 ^a^	6.73 ± 0.06 ^b^	6.68 ± 0.03 ^bc^	6.45 ± 0.02 ^bc^	6.22 ± 0.04 ^bc^	0.0392
CAR, um^2^	87.14 ± 0.043 ^a^	78.98 ± 0.12 ^b^	76.92 ± 0.06 ^c^	74.14 ± 0.16 ^d^	73.93 ± 0.05 ^d^	0.0001
RCN, um^2^	13.51 ± 0.28 ^a^	11.75 ± 0.12 ^b^	11.52 ± 0.03 ^b^	11.5 ± 0.05 ^b^	11.35 ± 0.07 ^b^	0.0006

Results are reported as means ± standard error of 3 groups per treatment (*n* = 3). Different letters (abcd) indicate significant differences (*p* < 0.05). Abbreviations: HAR (hepatocyte area), CAR (hepatocyte cytoplasm area), NAR (hepatocyte nucleus area), RCN (ratio of cytoplasmic area and nuclear area of the hepatocyte).

**Table 8:** Histomorphology of the kidney in *O. niloticus* fed diets supplemented with O. vulgare essential oil for 8 weeks and subsequently exposed to malathion-induced stress for 96 h.

Parameters	OEO Levels (g kg^−1^)					*p*-Value
	0 (Control)	0.75	1.50	2.25	3.00	
Before exposure						
GA, µm^2^	1742.37 ± 17.52 ^c^	1775.18 ± 9.57 ^c^	1886.52 ± 28.98 ^b^	1965.44 ± 18.49 ^a^	1928.24 ± 21.27 ^ab^	0.0043
DT-TA, µm^2^	221.61 ± 6.48 ^c^	271.4 ± 1.33 ^b^	279.95 ± 8.02 ^b^	348.52 ± 6.91 ^a^	346.82 ± 4.55 ^a^	0.0001
DT-LA, µm^2^	6.55 ± 0.54 ^d^	12.06 ± 1.44 ^cd^	14.57 ± 1.25 ^bc^	18.53 ± 0.99 ^b^	25.88 ± 0.64 ^a^	0.0002
DT-TWA, µm^2^	215.06 ± 5.68 ^a^	263.03 ± 1.01 ^a^	272.43 ± 5.86 ^b^	317.89 ± 3.60 ^b^	320.94 ± 0.82 ^b^	0.0001
DT-NN	6.61 ± 0.29 ^c^	10.88 ± 0.06 ^ab^	9.66 ± 0.13 ^b^	11.45 ± 0.50 ^a^	12.02 ± 0.12 ^a^	0.0055
PT-TA, µm^2^	246.12 ± 7.82 ^b^	269.57 ± 6.66 ^b^	336.83 ± 8.98 ^ab^	406.09 ± 9.99 ^a^	424.87 ± 6.74 ^a^	0.0312
PT-LA, µm^2^	10.08 ± 0.64 ^c^	17.89 ± 1.65 ^bc^	20.606 ± 1.57 ^bc^	26.81 ± 5.90 ^ab^	38.67 ± 0.04 ^a^	0.0002
PT-TWA, µm^2^	236.87 ± 7.46 ^c^	255.62 ± 7.21 ^bc^	286.348 ± 10.53 ^bc^	379.52 ± 4.90 ^a^	386.20 ± 6.18 ^ab^	0.0001
PT-NN	8.45 ± 0.252 ^c^	11.13 ± 0.84 ^b^	11.20 ± 0.20 ^b^	12.58 ± 0.13 ^ab^	13.65 ± 0.09 ^a^	0.0055
After exposure						
GA, µm^2^	1472.45 ± 27.74 ^c^	1658.78 ± 54.08 ^b^	1742.38 ± 55.96 ^b^	1915.92 ± 19.81 ^a^	1921.01 ± 22.89 ^a^	0.0202
DT-TA, µm^2^	586.11 ± 1.47 ^a^	399.17 ± 2.50 ^b^	385.16 ± 7.96 ^bc^	347.49 ± 0.64 ^bc^	353.63 ± 0.28 ^c^	0.0001
DT-LA, µm^2^	49.77 ± 0.43 ^a^	39.36 ± 5.02 ^ab^	28.40 ± 1.55 ^bc^	28.05 ± 0.61 ^bc^	21.06 ± 1.85 ^c^	0.0069
DT-TWA, µm^2^	537.24 ± 6.28 ^a^	329.71 ± 5.68 ^b^	350.25 ± 9.02 ^b^	327.40 ± 7.33 ^b^	326.07 ± 0.49 ^b^	0.0001
DT-NN	8.30 ± 0.18 ^c^	9.04 ± 0.16 ^bc^	10.52 ± 0.09 ^ab^	11.05 ± 0.19 ^a^	11.08 ± 0.13 ^a^	0.0001
PT-TA, µm^2^	629.44 ± 4.27 ^a^	499.57 ± 5.88 ^b^	479.31 ± 8.75 ^bc^	421.34 ± 5.88 ^d^	455.60 ± 7.28 ^cd^	0.0277
PT-LA, µm^2^	59.66 ± 7.06 ^a^	54.72 ± 0.57 ^ab^	51.33 ± 3.03 ^ab^	45.33 ± 2.90 ^ab^	33.50 ± 3.38 ^b^	0.0069
PT-TWA, µm^2^	463.35 ± 9.55 ^a^	461.14 ± 3.21 ^a^	445.29 ± 3.04 ^a^	355.62 ± 1.08 ^b^	298.32 ± 4.17 ^c^	0.0001
PT-NN	10.88 ± 0.18 ^b^	11.33 ± 0.27 ^b^	13.52 ± 0.14 ^a^	14.50 ± 0.03 ^a^	14.05 ± 0.12 ^a^	0.0012

Results are reported as means ± standard error of 3 groups per treatment (*n* = 3). Different letters (abcd) indicate significant differences (*p* < 0.05). Abbreviations: GA (glomerular area); PT-TA (proximal tubular area); PT-LA (proximal tubular luminal area); PT-TWA (proximal tubular wall area); PT-NN (number of nuclei in proximal tubules); DT-TA (distal tubular area); DT-LA (distal tubular luminal area); DT-TWA (distal tubular wall area); DT-NN (number of nuclei in distal tubules).

## Data Availability

Data are contained within the article. The supportive data of the findings of this study are available from the corresponding author upon request.

## Supplementary Materials

The following supporting information can be downloaded at: https://www.mdpi.com/article/10.3390/biology15141117/s1, Figure S1 (refs. [51,95,96] are cited in Supplementary Materials): Concentration–response curve from an independent bioassay fitted using a Probit model to estimate the 96 h LC50 of malathion in juvenile Nile tilapia (*Oreochromis niloticus*). Points represent the observed mortality proportion at 96 h for each evaluated concentration included in the fit. The solid line represents the fitted Probit model. The dashed horizontal line indicates 50% mortality, and the dashed vertical line indicates the estimated LC50-96 h value. The shaded area represents the 95% confidence interval. The Probit model was fitted using the natural logarithm of malathion concentration; the 0 mg L^−1^ control was excluded from the logarithmic fit because ln(0) is undefined; Figure S2: PCA loading plot showing the contribution of all variables to the first two principal components before the malathion challenge, after Z-score standardisation. PC1 and PC2 explained 65.16% and 12.00% of the total variance, respectively. Loadings were calculated as correlations between Z-score-transformed variables and principal component scores; Figure S3: PCA loading plot showing the contribution of all variables to the first two principal components after the malathion challenge, after Z-score standardisation. PC1 and PC2 explained 63.86% and 12.49% of the total variance, respectively. Loadings were calculated as correlations between Z-score-transformed variables and principal component scores; Table S1. PCA loadings of growth, metabolic, antioxidant, histological and gene expression variables contributing to PC1 and PC2 before malathion exposure; Table S2: PCA loadings of metabolic, antioxidant, histological and gene expression variables contributing to PC1 and PC2 after the malathion challenge.

## Abbreviations

The following abbreviations are used in this manuscript:

ABS	Absorbance
AFI	Apparent feed intake
ALP	Alkaline phosphatase
ALT	Alanine aminotransferase
ANOVA	Analysis of variance
ARE	Antioxidant response element
AST	Aspartate aminotransferase
BCG	Bromocresol green
BHT	Butylated hydroxytoluene
BIL	Bilirubin
Bp	Base pairs
CAR	Hepatocyte cytoplasm area
CAT	Catalase enzyme
cDNA	Complementary DNA
CF	Condition factor
CHOD-PAP	Cholesterol oxidase–phenol aminophenazone method
CHOL	Cholesterol
CREA	Creatinine
DB-5MS	5%-phenyl-methylpolysiloxane capillary column
DE	Digestible energy
DO	Dissolved oxygen
DT-LA	Distal tubular luminal area
DT-NN	Number of nuclei in distal tubules
DT-TA	Distal tubular area
DT-TWA	Distal tubular wall area
EI	Electron ionisation
FCR	Feed conversion ratio
FE	Feed efficiency
FL	Final length
FW	Final body weight
GA	Glomerular area
GC-MS	Gas chromatography–mass spectrometry
GLDH	Glutamate dehydrogenase
GLU	Glucose
GOD-PAP	Glucose oxidase–phenol aminophenazone method
*gpx*	Glutathione peroxidase gene
GPx	Glutathione peroxidase enzyme activity
GPO-PAP	Glycerol phosphate oxidase–phenol aminophenazone method
H&E	Haematoxylin and eosin
HAR	Hepatocyte area
HPLC	High-performance liquid chromatography
INT	Iodonitrotetrazolium chloride
Keap1	Kelch-like ECH-associated protein 1
*keap1*	Kelch-like ECH-associated protein 1 gene
LC50	Median lethal concentration
MDA	Malondialdehyde
MS	Mass spectrometry
NADPH	Nicotinamide adenine dinucleotide phosphate
NAR	Hepatocyte nucleus area
NFE	Nitrogen-free extract
*nrf2*	Nuclear factor erythroid 2-related factor 2 gene
Ns	Not significant
OEO	*Origanum vulgare* essential oil
PCA	Principal component analysis
PC1	Principal component 1
PC2	Principal component 2
PER	Protein efficiency ratio
PERMANOVA	Permutational multivariate analysis of variance
PERMDISP	Permutational analysis of multivariate dispersions
PT-LA	Proximal tubular luminal area
PT-NN	Number of nuclei in proximal tubules
PT-TA	Proximal tubular area
PT-TWA	Proximal tubular wall area
PTFE	Polytetrafluoroethylene
qPCR	Quantitative polymerase chain reaction
RCN	Cytoplasm-to-nucleus area ratio
RI exp.	Experimental retention index
RNA	Ribonucleic acid
ROS	Reactive oxygen species
RT	Retention time
RT-qPCR	Reverse transcription quantitative polymerase chain reaction
SGR	Specific growth rate
SOD	Superoxide dismutase
TBARS	Thiobarbituric acid reactive substances
TG	Triglycerides
TIC	Total ion chromatogram
TP	Total protein
UREA	Urea
UTEQ	Universidad Técnica Estatal de Quevedo
WG	Weight gain
