# Supplementary material for: Nrf2-Linked Antioxidant and Metabolic Modulation by Dietary Origanum vulgare Essential Oil in Nile Tilapia Under Organophosphate Stress
Source: Biology (Basel). 2026 Jul 10;15(14):1117. doi: 10.3390/biology15141117 (PMC13403801; doi:10.3390/biology15141117)
Supplement: Supplementary file 1 [file biology-15-01117-s001.zip › Figure S1.pdf]

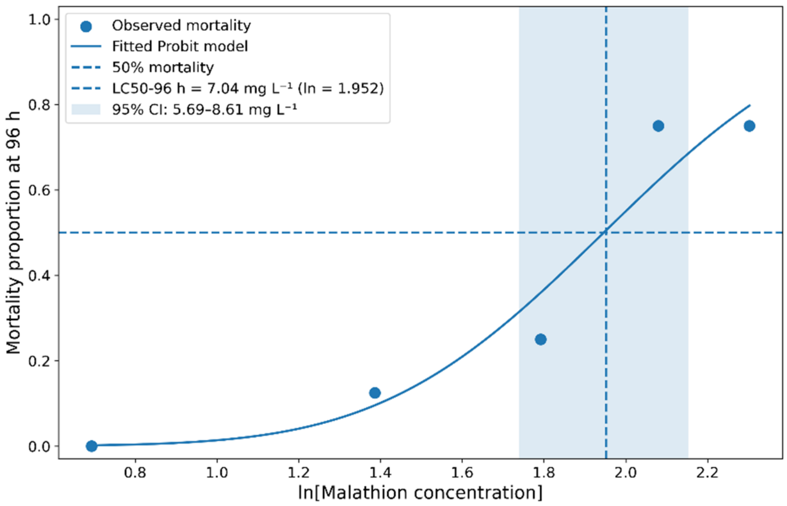

Figure S1. Concentration–response curve from an independent bioassay fitted using a Probit model to estimate the 96 h LC50 of malathion in juvenile Nile tilapia (*Oreochromis niloticus*). Points represent the observed mortality proportion at 96 h for each evaluated concentration included in the fit. The solid line represents the fitted Probit model. The dashed horizontal line indicates 50% mortality, and the dashed vertical line indicates the estimated LC50-96 h value. The shaded area represents the 95% confidence interval. The Probit model was fitted using the natural logarithm of malathion concentration; the 0 mg L^{-1} control was excluded from the logarithmic fit because ln(0) is undefined.

Supplementary Method

a) Determination of the 96 h LC_{50} of malathion in juvenile Nile tilapia

Before the main exposure assay, an independent concentration–response bioassay was conducted to estimate the 96 h median lethal concentration (LC_{50}-96 h) of malathion in juvenile Nile tilapia (*Oreochromis niloticus*). The bioassay was performed following the general approach of acute toxicity tests in fish, in which organisms are exposed to increasing concentrations of the compound for 96 h and cumulative mortality is used to estimate the LC_{50}-96 h [51].

The juveniles used in the bioassay had an average body weight of 23.4 ± 0.73 g. Before the assay, fish were acclimated for one week under controlled laboratory conditions in dechlorinated and aerated water. During this period, fish were fed a basal diet and maintained under suitable water-quality conditions for the species. Before the start of exposure, fish were fasted for 24 h. The bioassay included a control group without malathion exposure and five increasing nominal concentrations of the compound. The evaluated concentrations were 0, 2, 4, 6, 8 and 10 mg L^{-1}, where 0 mg L^{-1} corresponded to the control group. For each concentration, three replicate tanks were used, with 10 fish per tank, for a total of 30 fish per concentration. Fish were randomly distributed among the experimental tanks.

The exposure was maintained for 96 h under a semi-static system, in accordance with the generally accepted design for acute fish assays [51]. Water and malathion solutions were renewed every 24 h using freshly prepared solutions at the corresponding nominal concentrations. Fish were not fed during the bioassay. The physicochemical parameters of the water, including temperature, dissolved oxygen, pH and ammonia/ammonium, were monitored daily and maintained within suitable ranges for juvenile Nile tilapia, following general water-quality control criteria for aquatic assays [51,95].

Mortality was recorded at 24, 48, 72 and 96 h of exposure, according to the recommended time schedule for acute toxicity tests in fish [51]. Fish were considered dead when opercular movements were absent and no response to gentle tactile stimulation was observed. Dead fish were immediately removed from the tanks to avoid deterioration of water quality. At the end of 96 h, cumulative mortality was calculated for each malathion concentration. Cumulative mortality data at 96 h were analysed using the Probit method to estimate the LC_{50}-96 h and its 95% confidence interval [96]. The model was fitted using the natural logarithm of the nominal malathion concentrations; the 0 mg L^{-1} control was excluded from the logarithmic fit because ln(0) is undefined. The Probit analysis estimated a 96 h LC50 of 7.04 mg L^{-1}, with a 95% confidence interval of 5.69–8.61 mg L^{-1}.
