# Supplementary material for: Nrf2-Linked Antioxidant and Metabolic Modulation by Dietary Origanum vulgare Essential Oil in Nile Tilapia Under Organophosphate Stress
Source: Biology (Basel). 2026 Jul 10;15(14):1117. doi: 10.3390/biology15141117 (PMC13403801; doi:10.3390/biology15141117)
Supplement: Supplementary file 1 [file biology-15-01117-s001.zip › Figure S2.pdf]

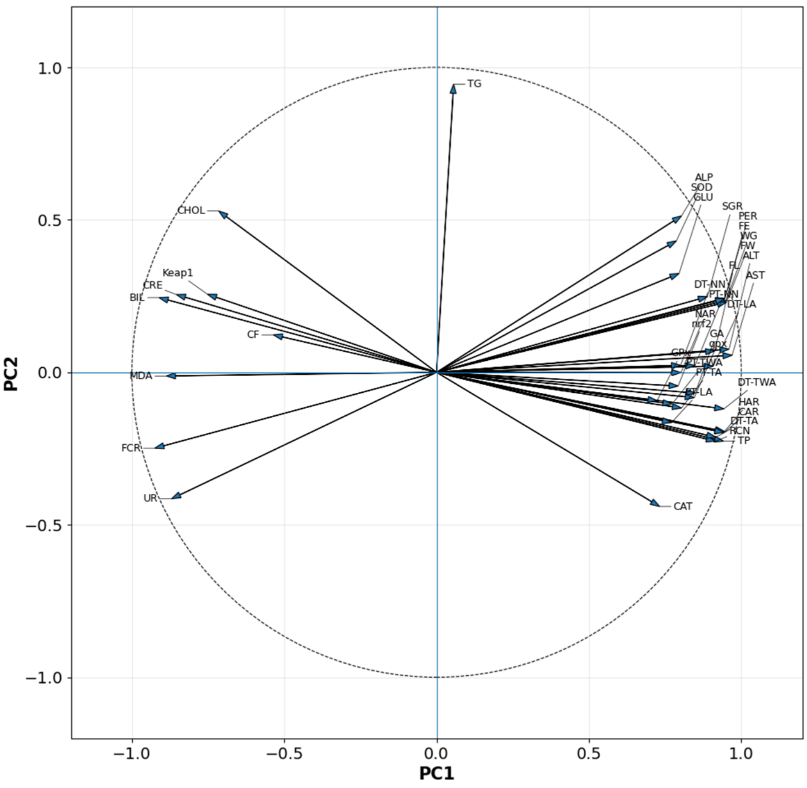

Figure S2. PCA loading plot showing the contribution of all variables to the first two principal components before the malathion challenge, after Z-score standardisation. PC1 and PC2 explained 65.16% and 12.00% of the total variance, respectively. Loadings were calculated as correlations between Z-score-transformed variables and principal component scores.

Note: Abbreviations: FW (Final body weight), FL (Final length), WG (Weight gain), SGR (Specific growth rate), CF (Condition factor), FCR (Feed conversion ratio), FE (Feed efficiency), PER (Protein efficiency ratio), GLU (Glucose), CHOL (Cholesterol), TG (Triglycerides), TP (Total protein), AST (Aspartate aminotransferase), ALT (Alanine aminotransferase), ALP (Alkaline phosphatase), SOD (Superoxide dismutase enzyme), CAT (Catalase enzyme), GPx (Glutathione peroxidase enzyme), MDA (Malondialdehyde), HAR (Hepatocyte area), NAR (Hepatocyte nucleus area), CAR (Hepatocyte cytoplasm area), RCN (Cytoplasm-to-nucleus area ratio), GA (Glomerular area), DT-TA (Distal tubular area), DT-LA (Distal tubular luminal area), DT-TWA (Distal tubular wall area), DT-NN (Number of nuclei in distal tubules), PT-TA (Proximal tubular area), PT-LA (Proximal tubular luminal area), PT-TWA (Proximal tubular wall area), PT-NN (Number of nuclei in proximal tubules), n*rf2* (Nuclear factor erythroid 2-related factor 2 gene), *gpx* (Glutathione peroxidase gene), and *keap1* (Kelch-like ECH-associated protein 1 gene).
