# Supplementary material for: Nrf2-Linked Antioxidant and Metabolic Modulation by Dietary Origanum vulgare Essential Oil in Nile Tilapia Under Organophosphate Stress
Source: Biology (Basel). 2026 Jul 10;15(14):1117. doi: 10.3390/biology15141117 (PMC13403801; doi:10.3390/biology15141117)
Supplement: Supplementary file 1 [file biology-15-01117-s001.zip › Table S1.pdf]

Table S1. PCA loadings of growth, metabolic, antioxidant, histological and gene expression variables contributing to PC1 and PC2 before malathion exposure.

Variable	PC1_loading	PC2_loading
WG	0.984380501	0.244366667
FW	0.985698239	0.238393032
PER	0.978957215	0.251057432
FE	0.979337601	0.249485863
AST	1.004172975	0.057813135
TP	0.975181802	-0.234428803
HAR	0.977907913	-0.200378144
CAR	0.976558929	-0.204631193
UR	-0.900396712	-0.429128536
ALT	0.993770171	0.078668006
FCR	-0.957051110	-0.257344961
ALP	0.831708151	0.531213150
DT-TWA	0.976340669	-0.123865355
TG	0.057483355	0.978678811
BIL	-0.943044080	0.254033327
RCN	0.946877315	-0.234281985
DT-TA	0.949006143	-0.220122183
SGR	0.920583235	0.256415183
FL	0.944949420	0.072082418
DT-LA	0.939609550	0.020558644
SOD enzyme	0.815100584	0.445878695
CRE	-0.883818614	0.263582090
CHOL	-0.739706971	0.547818716
MDA	-0.917920792	-0.012572914
GLU	0.823898823	0.335182999
CAT enzyme	0.758140424	-0.454979628
GA	0.878899964	-0.070725560
gpx gene	0.876206710	-0.086375635
PT-NN	0.877859162	0.023048495
PT-TA	0.832587863	-0.121024211
DT-NN	0.829340668	0.024104299
NAR	0.829662311	-0.001190820
keap1 gene	-0.778776616	0.264645112
nrf2 gene	0.821094352	-0.046554285
PT-LA	0.797985505	-0.170919238
PT-TWA	0.800092887	-0.107399476
GPx enzyme	0.749256790	-0.096185458
CF	-0.554821922	0.126891055

Note: Abbreviations: FW (Final body weight), FL (Final length), WG (Weight gain), SGR (Specific growth rate), CF (Condition factor), FCR (Feed conversion ratio), FE (Feed efficiency), PER (Protein efficiency ratio), GLU (Glucose), CHOL (Cholesterol), TG (Triglycerides), TP (Total protein), AST (Aspartate aminotransferase), ALT (Alanine

aminotransferase), ALP (Alkaline phosphatase), SOD (Superoxide dismutase enzyme), CAT (Catalase enzyme), GPx (Glutathione peroxidase enzyme), MDA (Malondialdehyde), HAR (Hepatocyte area), NAR (Hepatocyte nucleus area), CAR (Hepatocyte cytoplasm area), RCN (Cytoplasm-to-nucleus area ratio), GA (Glomerular area), DT-TA (Distal tubular area), DT-LA (Distal tubular luminal area), DT-TWA (Distal tubular wall area), DT-NN (Number of nuclei in distal tubules), PT-TA (Proximal tubular area), PT-LA (Proximal tubular luminal area), PT-TWA (Proximal tubular wall area), PT-NN (Number of nuclei in proximal tubules), n*rf2* (Nuclear factor erythroid 2-related factor 2 gene), *gpx* (Glutathione peroxidase gene), and *keap1* (Kelch-like ECH-associated protein 1 gene).
