# Supplementary material for: Nrf2-Linked Antioxidant and Metabolic Modulation by Dietary Origanum vulgare Essential Oil in Nile Tilapia Under Organophosphate Stress
Source: Biology (Basel). 2026 Jul 10;15(14):1117. doi: 10.3390/biology15141117 (PMC13403801; doi:10.3390/biology15141117)
Supplement: Supplementary file 1 [file biology-15-01117-s001.zip › Table S2.pdf]

Table S2. PCA loadings of metabolic, antioxidant, histological and gene expression variables contributing to PC1 and PC2 after the malathion challenge.

Variable	PC1_loading	PC2_loading
CAR	0.980950914	0.116464798
HAR	0.982822198	0.092485773
CRE	0.911256044	-0.357221249
ALP	0.593269816	0.769496562
UR	0.891110356	-0.381442918
DT-TA	0.927000711	0.250356247
ALT	0.690905425	0.664358774
gpx gene	-0.947805462	0.137571855
keap1 gene	0.944815258	-0.114689093
nrf2 gene	-0.939660033	0.131700393
AST	0.714092317	0.620367148
GA	-0.943156425	0.067092465
DT-TWA	0.842267526	0.421043129
DT-NN	-0.909005034	0.172204953
PT-TA	0.919011235	0.105432296
RCN	0.847453078	0.349333407
PT-NN	-0.843978467	0.333063745
GPx enzyme	-0.813145743	0.376404534
BIL	0.871859033	0.191155262
CAT enzyme	-0.859113032	-0.147391216
TP	-0.304423496	0.801277380
DT-LA	0.856757378	-0.003526332
PT-TWA	0.801730935	-0.267823733
CHOL	0.819507422	-0.049758171
MDA	0.791363730	0.066664058
SOD enzyme	-0.790514896	-0.044091214
RPL	-0.713687311	0.296236254
SUP	-0.701169963	0.290889966
GLU	0.654966080	-0.140551669
PT-LA	0.573356112	-0.128347023
NAR	0.005111649	-0.569702943
TG	-0.005698760	0.510577061

Note: Abbreviations: GLU (Glucose), CHOL (Cholesterol), TG (Triglycerides), TP (Total protein), AST (Aspartate aminotransferase), ALT (Alanine aminotransferase), ALP (Alkaline phosphatase), SOD (Superoxide dismutase enzyme), CAT (Catalase enzyme), GPx (Glutathione peroxidase enzyme), MDA (Malondialdehyde), HAR (Hepatocyte area), NAR (Hepatocyte nucleus area), CAR (Hepatocyte cytoplasm area), RCN (Cytoplasm-to-nucleus area ratio), GA (Glomerular area), DT-TA (Distal tubular area), DT-LA (Distal tubular luminal area), DT-TWA (Distal tubular wall area), DT-NN (Number of nuclei in distal tubules), PT-TA (Proximal tubular area), PT-LA (Proximal tubular luminal area), PT-TWA (Proximal tubular wall area), PT-NN (Number of nuclei in proximal tubules), SUP (Survival), n*rf2* (Nuclear factor erythroid 2-related factor 2

gene), *gpx* (Glutathione peroxidase gene), and *keap1* (Kelch-like ECH-associated protein 1 gene).
